# In vitro and in vivo antitumor activity of Laz-expressing Salmonella Typhimurium YB1 via optimized secretion and regulation

**DOI:** 10.1186/s13568-026-02057-x

**Published:** 2026-05-15

**Authors:** Yahia I. Abouelenen, Radwa M. Ramzy, Alshaymaa Amin Zaki El-Bahy, Christian U. Riedel, Khaled M. Abou-Aisha, Manar M. Mansour

**Affiliations:** 1 Department of Microbiology and Immunology, Faculty of Pharmacy and Biotechnology, The German University in Cairo (GUC), Main Entrance fifth settlement, Cairo, Egypt; 2 School of Life and Medical Sciences, University of Hertfordshire, Hosted by global academic foundation, Cairo, Egypt; 3 Microbial Biotechnology, Biology Department, Ulm University (UULM), Albert-Einstein-Allee 11, 89069 Ulm, Germany; 4 Department of Pharmaceutical Biology and Microbiology, Faculty of Pharmacy and Biotechnology, The German University in Cairo (GUC), Main Entrance fifth settlement, Cairo, Egypt

**Keywords:** Bacterial cancer therapy, *Salmonella* Typhimurium YB1, Laz, Tumor targeting, Ehrlich carcinoma, Protein delivery

## Abstract

*Salmonella enterica* serovar Typhimurium strain YB1 is a genetically engineered obligate anaerobe with strong tumor-targeting potential. In this study, we evaluated YB1 as a delivery platform for Laz, a lipidated azurin-derived protein, using three secretion strategies: a native lipoprotein signal peptide (SPase II; YDL), a Sec-dependent secretory signal peptide (SPase I; YDN), and a Type III secretion signal (YDP). Laz expression was controlled by the aTc-inducible Pxyl/tet promoter on a pET29a backbone. To eliminate the confounding effects of metabolic burden, we constructed a TetR-matched control plasmid (named YC*) to be used as a comparator. Compared with YC*, Laz-expressing YB1 strains significantly reduced the viability of MCF-7 cells: YDL (50.69 ± 5.51%, *p* < 0.001), YDN (52.05 ± 4.69%, *p* < 0.001), and YDP (51.03 ± 3.92%, *p <* 0.001). This effect was dependent on induction conditions, with stronger responses observed at higher aTc concentrations. In vivo evaluation using the Ehrlich carcinoma mouse model revealed increased tumor necrosis and reduced mitotic activity in bacterially-treated groups. Among the constructs, YDL exhibited higher Laz expression and export, which was associated with more pronounced effects in vitro and consistent trends in vivo. Collectively, these findings support the feasibility of YB1-mediated Laz delivery as a bacterial delivery platform and highlight the importance of matched regulatory architecture in the design of engineered bacterial therapeutics. This study provides a functional evaluation of Laz-associated cytotoxic effects in the YB1 system while indicating areas for further investigation.

**Supplementary Information:**

The online version contains supplementary material available at 10.1186/s13568-026-02057-x.

## Introduction

The use of biological agents, such as viruses and bacteria, for targeted cancer therapy has garnered growing interest due to their unique capabilities to home to tumors, replicate in hypoxic environments, and deliver therapeutic payloads directly into malignant tissues (Fukuhara et al. 2016; Krzykawski 2015; Ryan et al. 2009; Song et al. 2018). While viral vectors have advanced to clinical use (Kudlay and Svistunov 2022), their application in cancer therapy faces limitations related to immune clearance, toxicity, and restricted tumor penetration (Alemany et al. 2000; Gong et al. 2016; Hirasawa et al. 2003; Krzykawski 2015; Reid et al. 2002; Russell et al. 2014; Toley and Forbes 2012; White et al. 2008). In contrast, certain bacterial species, especially facultative anaerobes like *Salmonella enterica* serovar Typhimurium, offer significant advantages, including deeper tumor infiltration, longer circulation times, and ease of genetic engineering (Clairmont et al. 2000; Krzykawski 2015; Toso et al. 2002).

Among *Salmonella* strains, YB1 has emerged as a promising vector due to its engineered safety and tumor selectivity (Yu et al. 2012). YB1 is a derivative of the *aroA*-deficient SL7207 strain, further modified by placing the essential *asd* gene under hypoxia-inducible control (PpepT promoter) and repressing its expression under normoxic conditions (PsodA antisense promoter). This engineering renders YB1 replication-competent only in hypoxic tumor environments, thereby reducing off-target effects and improving its biosafety profile. YB1 has been shown to colonize tumor tissues efficiently while being rapidly cleared from normal tissues and systemic circulation (Yu et al. 2012, 2015).

The potential of using *S.* Typhimurium as a delivery vehicle extends beyond its tumor-colonizing ability (Kasinskas and Forbes 2006; Leschner and Weiss 2010; Leschner et al. 2009; Wang et al. 2016) to its capacity to continuously produce and secrete therapeutic agents in situ. This has inspired efforts to load *Salmonella* with cytotoxic genes or proteins, enabling prolonged exposure of cancer cells to tumoricidal molecules. One such molecule is lipidated azurin (Laz), a surface-exposed lipoprotein from *Neisseria meningitides* (Gotschlich and Seiff 1987) with well-documented antitumor effects. Laz, like other azurins, has been reported to induce apoptosis in cancer cells by stabilizing the tumor suppressor p53, halting cell cycle progression, and modulating pathways involved in proliferation, angiogenesis, and metastasis (Bernardes et al. 2014; Chaudhari et al. 2007; Hu et al. 2023; Punj et al. 2004; Yamada, Goto, Punj, Zaborina, Chen, et al. 2002a; Yamada et al. 2009). Its unique H.8 epitope and SPase II-cleaved signal peptide distinguish it structurally and functionally from other azurin homologs, such as Paz from *Pseudomonas aeruginosa* (Gotschlich and Seiff 1987; Hashimoto et al. 2015; Hong et al. 2006).

Our previous work demonstrated the successful expression of Laz in VNP20009 (Mansour et al. 2020), another attenuated *Salmonella* strain, under the control of a hypoxia-inducible promoter. Laz localized to the bacterial membrane and conferred enhanced cytotoxicity to the recombinant strain in vitro. However, protein surface exposure alone may limit therapeutic potential, prompting the current investigation into alternative export strategies.

The rationale of this study is to exploit the complementary mechanisms of tumor targeting by YB1 and the antitumor activity of Laz. As an obligate anaerobe, YB1 preferentially localizes to and proliferates within the hypoxic cores of solid tumors, where conventional therapies often show limited efficacy, thereby inducing localized tumor cell damage. However, since bacterial colonization is mostly restricted to these hypoxic regions. We therefore hypothesized that engineering YB1 to express and export Laz, as a secreted cytotoxic protein, would extend the therapeutic effect beyond the hypoxic niches to the more oxygenated peripheral tumor regions. This dual strategy was designed to enhance overall antitumor efficacy by combining spatially targeted bacterial colonization with broader paracrine cytotoxic activity.

Multiple secretion signals were introduced to explore different pathways for protein export. These included: (1) the native SPase II signal of Laz for outer membrane anchoring (strain YDL); (2) the NSP4 signal peptide (Han et al. 2017) cleaved by SPase I for secretion into the extracellular medium (YDN); and (3) type III secretion system (T3SS) signals from SopE and SptP (Konjufca et al. 2006, 2008) for host cytoplasmic delivery (YDE and YDP, respectively). Expression of each construct was driven by the chemically inducible Pxyl/tet promoter and evaluated in vitro against two-dimensional cultures of MCF-7 breast cancer cell line; and in vivo against Ehrlich ascites carcinoma (EAC) mouse model.

This work emphasizes the expression and export of Laz from the tumor-targeting YB1 strain and examines its delivery efficiency. By comparing distinct secretion routes and correlating them with cellular outcomes, this study lays a foundation for further optimization of bacterial vectors for targeted protein therapy in cancer.

## Materials and methods

### Bacterial strains and plasmids

The bacterial strains and plasmids used in this study are summarized in Table 1.

**Table 1:** Bacterial strains used in this study

Strain	Plasmid	Insert	Description and/or genotype	Source
*E. coli* DH10B	–	–	*F– mcrA Δ(mrr-hsdRMS-mcrBC) φ80lacZΔM15 ΔlacX74 recA1 endA1 araD139Δ(ara-leu)7697 galU galK λ– rpsL(StrR) nupG*	Thermo fisher scientific GmbH, Ulm, Germany
*S.* Typhimurium YB1	–	–	SL7207; Cm^R^; *∆asd::cm-PpepT-asd-PsodA*; obligate anaerobic *S.* Typhimurium	Kind gift from Prof. J. Huang (Univ. of Hong Kong)
YA	pUC19	–	Empty vector control strain; Amp^R^	This work
YC	pET29a	–	Empty vector control strain; Kan^R^	This work
YC*	pET29a	–	TetR–Pxyl/tet; Kan^R^	This work
YCL, YCN, YCE, YCP	pET29a	*laz-his*, etc.	TetR–Pxyl/tet–RBS–*laz* variant–*his-tag*; Kan^R^	This work
YDL, YDN, YDE, YDP	pET29a	*laz*, etc.	TetR–Pxyl/tet–RBS–*laz* variant–stop codon (no His tag); Kan^R^	This work

### Plasmid construction and genetic engineering

The *laz* gene (GenBank accession: NC_003112.2; locus tag: NMB_RS07975) was codon-optimized for *Salmonella* Typhimurium and synthesized along with three variants—*nsp4laz*, *sopElaz*, and *sptPlaz*—by Eurofins Scientific (Luxembourg); sequences used are provided in Supplementary Table S1. These constructs were designed to enable secretion of Laz through either signal peptidase I (SPase I), signal peptidase II (SPase II), or the type III secretion system (T3SS).

**Fig. 1:**
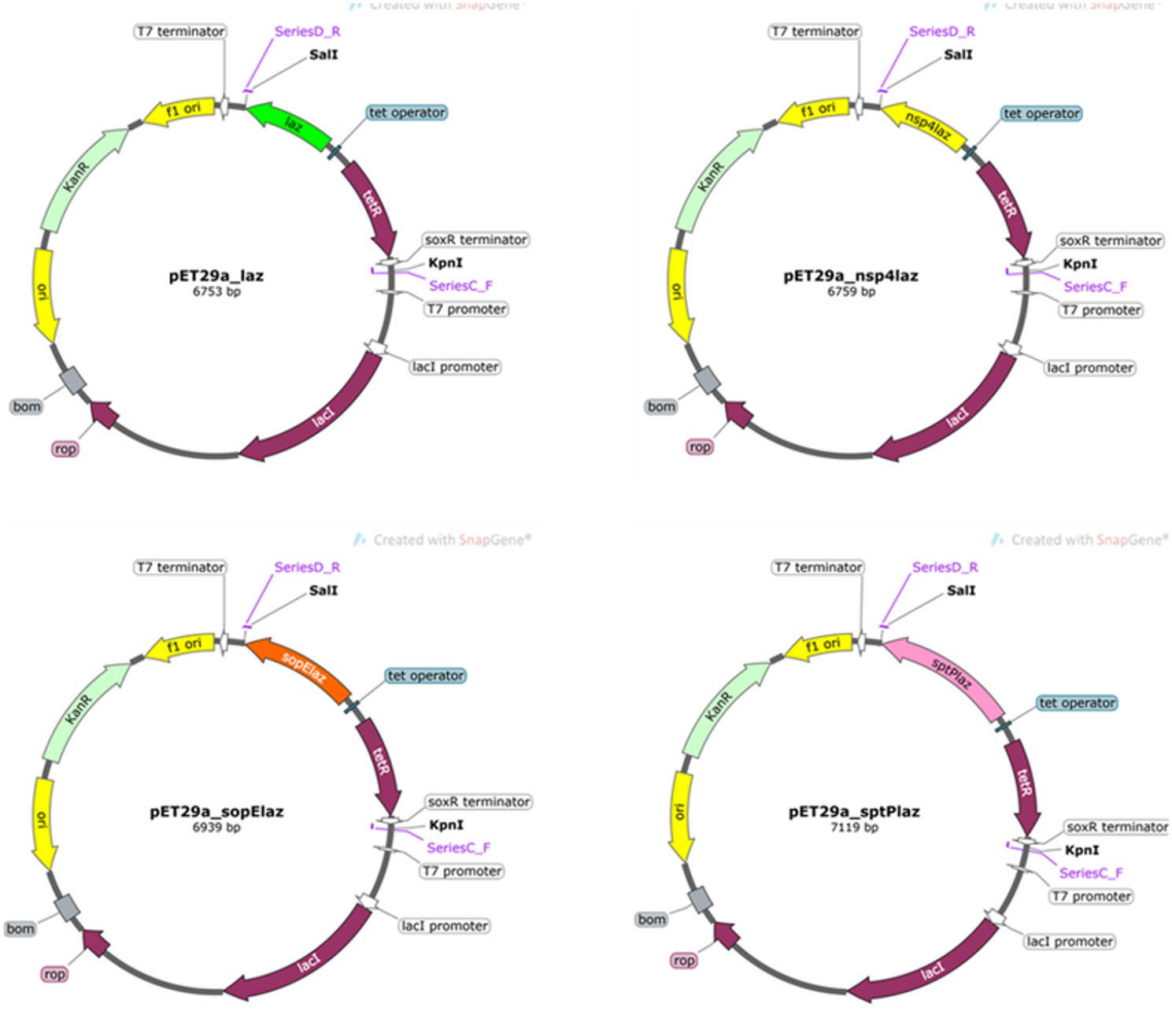
Vector maps of pET29a-*laz*, *nsp4laz*, *sopElaz*, and *sptPlaz*, excluding the C-terminal His-tag. The corresponding bacterial strains harboring these constructs are referred to as YDL, YDN, YDE and YDP in the subsequent experiments

An anhydrotetracycline (aTc)-inducible expression cassette containing the *tet*R repressor, Pxyl/tet promoter, and a ribosome binding site was amplified from pNZ-sg1594 (kindly provided by apl. Prof. C. Riedel, Ulm University, Germany). Each *laz* variant was inserted into pUC19 downstream of the promotor fragment using Gibson assembly (Supplementary Fig. S1), and subsequently cloned into the expression vector pET29a (Novagen, Germany) (Supplementary Fig. S2). For detection purposes, constructs were generated with (Supplementary Fig. S3) or without a C-terminal 6×His-tag (Fig. 1). All constructs were flanked by SoxR and T7 terminators to prevent interference from the vector backbone. The primers used for cloning are listed in Supplementary Table S2. Empty pET29a was used in the control strain YC, but was later replaced with another (pET29a-*tetR*) to create a more reliable comparison to the test strains. The latter was cloned similarly to the above mentioned pET29a plasmids minus the *laz*-derived portion.

Correct clones were verified by colony PCR and Sanger sequencing. Plasmids were introduced into *S.* Typhimurium YB1 via electroporation. The final recombinant strains are listed in Table 2.

**Table 2:** Summary of YB1-derived strains

Strain and vector backbone	Gene-of-interest	tetR	Strain acronym	
YB1-pET29a-	NA	−	YC	
	NA	+	YC*	
	Gene-of-interest	*tet*R	+ C-terminal His-tag	− C-terminal His-tag
	*laz*	+	YCL	YDL
	*nsp4laz*	+	YCN	YDN
	*sopElaz*	+	YCE	YDE
	*sptPlaz*	+	YCP	YDP

### Bacterial growth

All strains were cultured in Luria-Bertani (LB) medium with appropriate antibiotics. YB1-derived strains were supplemented with chloramphenicol (25 µg/mL) (Sigma Aldrich, Germany) and diaminopimelic acid (DAP; 50 µg/mL) (Alfa Aesar, USA). Overnight cultures were initiated from single colonies and incubated at 37 °C with shaking at 200 rpm until mid-exponential phase (OD_600_ = 0.4–0.6). For determination of viable cell counts, plating and enumeration of serially diluted cultures was done on LB agar plates with appropriate additives.

### Plasmid stability assay

To evaluate plasmid retention over ~ 50 generations, cultures were passaged every ~ 10 generations without antibiotic selection, based on 1000-fold dilutions. Colony-forming units (CFUs) were plated on selective (antibiotic^+^) and non-selective (antibiotic^−^) media to determine plasmid-bearing versus total viable counts, respectively. The percentage of plasmid-positive CFU to total CFU, indicated the degree of plasmid stability (%).

### Expression and export of laz

Crude cell lysates and culture supernatants were analyzed for Laz expression. Prior to preparation in Laemmli buffer, bacterial cultures were normalized to identical OD_600_ values to ensure equal bacterial biomass loading per lane. For supernatant samples, volumes corresponding to equivalent whole-culture OD_600_ values were collected for analysis of secreted Laz. Densitometric analysis using ImageJ software (National Institutes of Health, USA) was applied only to bands derived from samples resolved on the same membrane. For cell lysates, cultures were boiled and loaded directly. For supernatants, cultures were filtered (0.2 μm), and proteins were precipitated with trichloroacetic acid (TCA; 20% final concentration) (Carl Roth, Germany), washed in cold acetone, dried at 95 °C, and resuspended in Laemmli buffer for SDS-PAGE.

Protein detection was performed via western blotting using standard protocols (Bio-Rad Mini-PROTEAN and Trans-Blot Turbo systems). Membranes were blocked in 5% skim milk (in TBS-T buffer) and incubated with primary antibodies: anti-Lip/H.8 mAb 2C3 (Apicella et al. 1990) (a kind gift from Prof. R. Say). HRP-conjugated secondary antibodies (Rabbit Anti-Mouse IgG H&L; Abcam, UK) were used with SuperSignal™ West Femto substrate (Thermo Fisher Scientific GmbH, Germany) and visualized with an iBright™ imaging system (Thermo Fisher Scientific GmbH, Germany).

### Laz delivery into mammalian cells

HT-29 cells were infected with YB1 strains at a multiplicity of infection (MOI) of 100 and induced with 1000 ng/mL aTc. After 24 h of infection in antibiotic-free conditions, proteins were isolated from four fractions: extracellular bacteria (EB), intracellular bacteria (IB), extracellular secreted proteins (EP), and intracellular proteins (IP). Fractionation was performed using centrifugation and PBS washes, followed by Triton X-100 lysis of mammalian cells. TCA precipitation was used for protein concentration as described above. 

### Cell lines and culture conditions

HT-29 (ATCC HTB-38) and MCF-7 (ATCC HTB-22) cell lines were cultured in Dulbecco’s Modified Eagle’s Medium (DMEM) supplemented with 10% fetal bovine serum (FBS), 1% Penicillin-Streptomycin, and 2 mM L-glutamine (Sigma-Aldrich, Germany). Cells were maintained at 37 °C in a 5% CO_2_ incubator. Cell viability was assessed by trypan blue exclusion and light microscopy. The MCF-7 breast cancer cell line was selected for the primary in vitro experiments due to its expression of wild-type p53 (Leroy et al. 2014), a known molecular target of the Laz protein (Hashimoto et al. 2015; Yamada, Goto, Punj, Zaborina, Kimbara, et al. 2002b). HT-29 cells were used in preliminary experiments to optimize the protein delivery protocol and verify cellular uptake in mammalian cells. For in vivo evaluation, the Ehrlich ascites carcinoma (EAC) mouse model was used as a well-established transplantable tumor system for assessing antitumor activity.

### MTT cytotoxicity assay

Cytotoxicity was measured using the MTT assay. Cells were seeded in 96-well plates at 2 × 10^4^ cells/well (unless stated otherwise) and infected after 24 h. Bacterial cultures were prepared to a desired MOI (typically 100:1) using OD_600_ and a pre-established cfu: OD standard curve. Infection was allowed for two hours, after that, cells were washed and treated with a standard gentamicin (Carl Roth, Germany) treatment (50 µg/mL^−1^ h) to kill extracellular bacteria (Sharma and Puhar 2019). The maintenance phase after infection included culture media supplemented with kanamycin (Sigma-Aldrich, Germany), chloramphenicol, aTc (0–1000 ng/mL), and DAP. Post-infection, cell viability was assessed by incubating cells with MTT reagent (0.5 mg/mL (SERVA Electrophoresis GmbH, Germany) in PBS containing 10 µg/mL tetracycline (Sigma-Aldrich, Germany) for 4 h. Formazan crystals were dissolved in Dimethyl sulfoxide (DMSO) (Sigma-Aldrich, Germany) and absorbance was measured at 595 nm. Viability was expressed as a percentage relative to untreated controls.

### In vivo study on mice with Ehrlich Ascites Carcinoma

#### Animals

Thirty-five Swiss albino mice (aged 3–4 weeks, weighing 20–25 g) were obtained from *The Holding Company for Biological Products & Vaccines (VACSERA)*, Cairo, Egypt. Mice were acclimatized for three weeks in the animal house of the *Faculty of Pharmacy and Biotechnology*,* The German University in Cairo* (Cairo, Egypt). each experimental group was kept in a separate cage (five per cage) under controlled environmental conditions: temperature (25 ± 2 °C), relative humidity (60 ± 10%), and a 12 h light/dark cycle. Standard rodent chow and autoclaved water were provided *ad libitum* throughout the study. All experimental protocols were reviewed and approved by the *Ethics Committee for Animal Experimentation at the Faculty of Pharmacy*,* The German University in Cairo* (*CPH-2005-09-KAA*) in accordance with the *Guide for the Care and Use of Laboratory Animals* (“Guide for the Care and Use of Laboratory Animals,” 2011). Every effort was made to minimize animal suffering and to reduce the number of animals used.

#### Tumor induction

Ehrlich Ascites Carcinoma (EAC) cells were obtained from a donor Swiss albino mouse purchased from *The National Cancer Institute*,* Cairo*,* Egypt.* Ascitic fluid was harvested from the donor mouse, and viable EAC cells were counted and diluted in sterile phosphate-buffered saline (PBS) to a concentration of 1 × 10^6^ cells/mL. Each mouse in the tumor-bearing groups received a subcutaneous injection of 0.2 mL containing 2 × 10^5^ cells into the right hind thigh, as previously described (Gothoskar and Ranadive 1971). Female mice were used exclusively in this study because EAC cells are documented to exhibit higher initial proliferation rates and total cell counts in females compared to males (Vincent and Nicholls 1967). Tumors developed within seven days post-inoculation, and tumor growth was monitored until tumor volumes reached approximately 100–200 mm^3^. Tumor dimensions were measured using digital calipers, and tumor volumes were estimated according to the formula:

V=(a×b^2^)/2.

where *V* represents the tumor volume, *a* represents the longest tumor diameter (length) and *b* represents the shortest tumor diameter (width).

#### Bacterial preparation and infection of mice

All *Salmonella* Typhimurium strains were cultured overnight at 37 °C in LB broth supplemented with DAP and appropriate antibiotics (chloramphenicol, kanamycin). The optical density at 600 nm (OD₆₀₀) was measured and correlated to colony-forming units (cfu/mL) using a pre-established standard curve. Bacteria were harvested by centrifugation, washed twice, and resuspended in sterile PBS to the final working concentration for injection. Purity and viability were confirmed by plating serial dilutions on LB agar plates.

Twelve days after tumor inoculation, tumors reached the target volume, and mice were injected intravenously via the tail vein with 100 µL of bacterial suspension containing 5 × 10^7^ CFU (Yu et al. 2012, 2015). Two days later, mice received an intraperitoneal injection of 250 µL PBS containing aTc (1 µg/mL), and the treatment was repeated every other day to induce the expression of Laz (or its derivatives) (Hernandez-Abanto et al. 2006). Mice were sacrificed 23 days after bacterial injection, corresponding to a total experimental duration of 35 days from tumor inoculation.

#### Experimental design

Mice were randomly assigned into seven groups (*n* = 5 per group). Groups 1–5 were tumor-bearing groups receiving different treatments, while groups 6 and 7 were used as healthy controls (non-tumor bearing) for comparison of histopathology. The experimental groups are summarized in Table 3.

**Table 3:** Experimental design of animal study

Group	Tumor (+/-)	*n*	Treatment	Description
1	+	5	Sterile **PBS** (three times per week for 3 weeks)	Untreated control for tumor-bearing mice
2	+	5	YC* (YB1-pET29a-*tet*R)	Control strain
3	+	5	**YDL** (YB1-pET29a-*tet*R-laz)	Laz
4	+	5	**YDP** (YB1-pET29a-*tet*R-*sptPlaz*)	T3SS-injectable Laz
5	+	5	**YB1**	Parent strain
6	−	5	YC* (YB1-pET29a-*tet*R)	Control strain on healthy mice
7	−	5	Sterile PBS (three times per week for 3 weeks)	Untreated control for healthy mice

#### Tissue sampling

At the end of the treatment period, mice were euthanized under deep anesthesia induced by diethyl ether inhalation in accordance with the approved institutional animal ethics protocol. Tumor masses were carefully excised, and a portion of each tumor was fixed in 10% neutral buffered formalin for histopathological analysis.

#### Histopathological analysis

Tumor tissue samples were fixed in 10% neutral buffered formalin for 72 h. Tissues were then processed through a graded ethanol series, cleared in xylene, and embedded in paraffin wax. Section  (5 μm thick) were cut using a rotary microtome and mounted on glass slides. For general histological examination, sections were stained with Hematoxylin and Eosin (H&E) according to standard protocols (Culling 1974a, b, c). All slides were examined in a blinded manner by an experienced histopathologist.

#### Morphometric analysis

Morphometric assessment of tumor necrosis was performed on H&E-stained sections according to methods adopted from (Elgewelly et al. 2025; Mahmoud et al. 2022); at least six random non-overlapping fields per tumor section were captured using a Full HD microscopic imaging system (Leica Microsystems GmbH, Germany). The mean percentage of necrotic area per field was quantified using the Leica application module for histopathological analysis.

### Statistical analysis

Statistical analyses were performed using Microsoft Excel 2016 supplemented with the Real Statistics Resource Pack add-in. Data are presented as mean ± standard deviation (SD). Normality of the data distribution was assessed prior to parametric testing.

For comparisons involving multiple independent groups, including in vitro MTT cytotoxicity assays and morphometric analyses, one-way analysis of variance (ANOVA) followed by Tukey’s post-hoc test was applied. For longitudinal datasets, including tumor growth curves, plasmid stability measurements, and body weight monitoring, two-way repeated-measures ANOVA was used to evaluate the effects of treatment and time. When the assumption of sphericity was violated, Greenhouse–Geisser corrections were applied to adjust the p-values. Student’s unpaired two-tailed t-test was used only for independent pairwise comparisons where appropriate.

Potential outliers within each experimental group were assessed using Grubbs’ test (α = 0.05). When identified, outliers were excluded prior to statistical analysis. A *p* value < 0.05 was considered statistically significant.

## Results

### Plasmid stability and selection of the expression vector

**Fig. 2:**
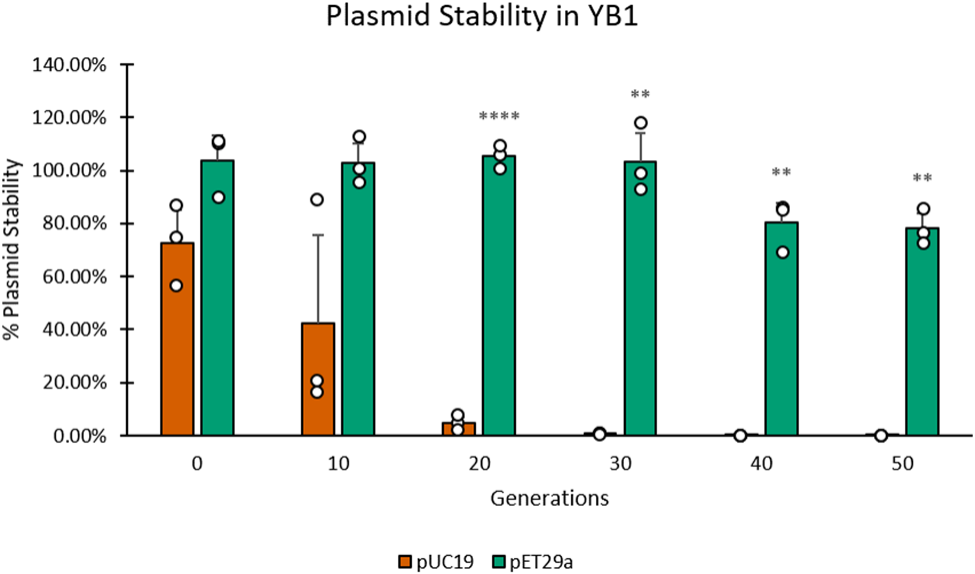
Plasmid retention in YB1 carrying pUC19 or pET29a was monitored over 50 generations. Data are presented as mean ± SD (*n* = 3). Statistical analysis was performed using a mixed two-way repeated measures ANOVA. Pairwise comparisons between plasmids at each generation were evaluated using Student’s t-test. Asterisks indicate significant differences compared with pUC19 (*p < 0.05, **p < 0.01, ***p < 0.001, **** *p* < 0.0001).

Initial tests using pUC19 revealed high plasmid instability in *S.* Typhimurium YB1 (Fig. 2), in agreement with previous reports (Gahan et al. 2007). Plasmid stability was assessed over 50 generations in strains carrying pUC19 or pET29a. A marked difference in plasmid retention was observed between the two vectors. Cells harboring pET29a maintained high plasmid stability throughout the experiment, with retention remaining above 78% after 50 generations, whereas pUC19 exhibited rapid plasmid loss, declining to nearly undetectable levels by generation 50. A mixed two-way repeated measures ANOVA revealed a significant effect of plasmid type on plasmid stability (F(1,4) = 687.48, *p* < 0.001), indicating that pET29a is significantly more stable than pUC19 during serial propagation.

### Expression, export, and delivery of laz

Western blot analysis confirmed the expression of Laz, NSP4Laz, and SptPLaz, but not SopELaz, in YB1-derived strains. The anti-Lip/H.8 mAb 2C3 as a primary antibody showed higher sensitivity compared to anti-His antibodies (Supplementary Fig. S4) which seemingly failed to detect potential truncated forms of SptPLaz detected by the former. Based on the location targeted by the antibody, this might suggest degradation in the C-terminus of the protein.

**Fig. 3:**
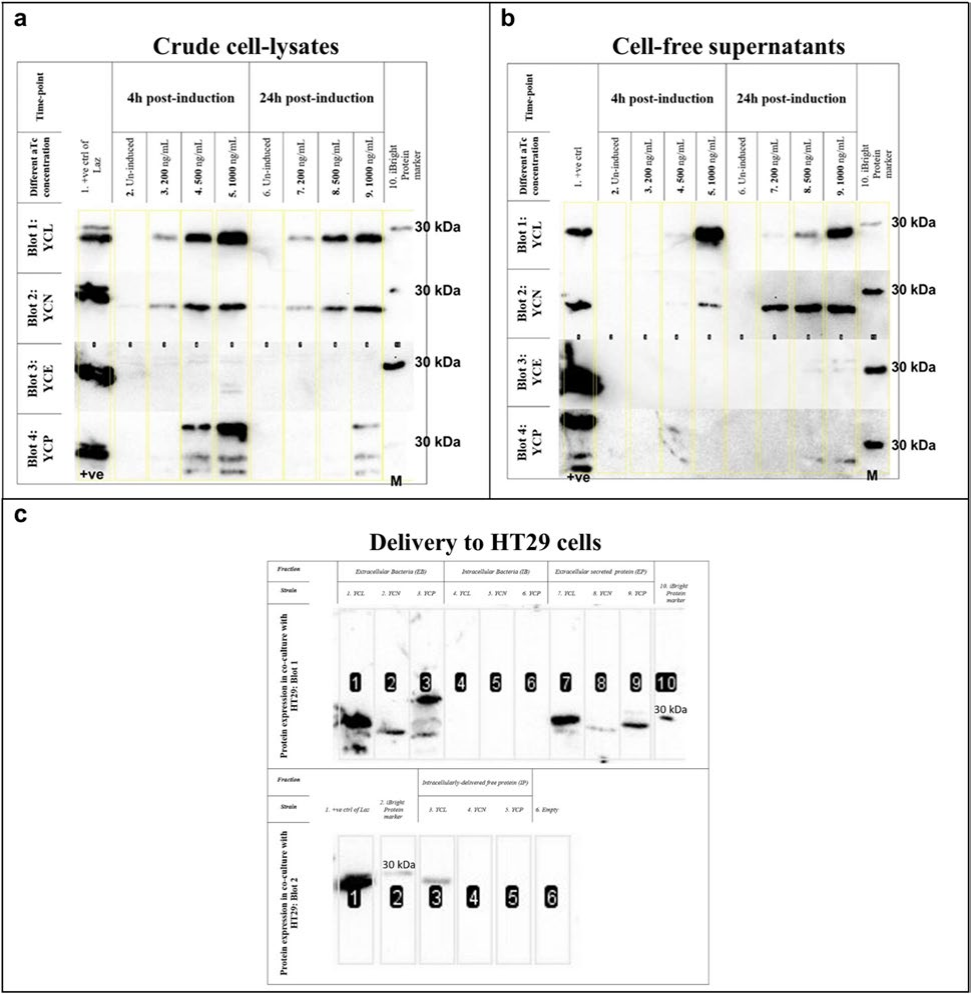
Western blots of Laz and its derivatives in bacteriological media and co-culture setting. Western blots of **a** crude cell lysates and **b** cell-free supernatants from Laz-producing YB1 cultures induced with various concentrations of anhydrotetracycline (aTc). *Blot 1*: YB1-pET29a-*laz-his* (YCL); *Blot 2*: YB1-pET29a-*nsp4laz-his* (YCN); *Blot 3*: YB1-pET29a-*sopElaz-his* (YCE); *Blot 4*: YB1-pET29a-*sptPlaz-his* (YCP). Lanes 2–5: samples taken 4 h post-induction; Lanes 6–9: samples taken 24 h post-induction. aTc concentrations: 0, 200, 500, and 1000 ng/mL. **c** Western blot analysis of Laz expression and delivery to HT-29 cells 24 h post-infection. Protein fractions include extracellular bacteria (EB), intracellular bacteria (IB), extracellular soluble protein (EP), and intracellularly delivered protein (IP)

Induction with increasing aTc concentrations enhanced protein expression and reduced bacterial growth (Supplementary Fig. S5), especially when added after cultures reached mid-exponential phase. Crude lysates showed more intense protein bands of Laz, NSP4Laz and SptPLaz at 4 h post-induction than at 24 h (Fig. 3a, Supplementary Fig. S6a). Laz and NSP4Laz were secreted into supernatants with differing kinetics: Laz secretion was inducer-dependent, whereas NSP4Laz exhibited time-dependent secretion (Fig. 3b, Supplementary Fig. S6b). SptPLaz secretion was weak but was improved under in vitro conditions mimicking the intestinal environment (Fig. 3b, Supplementary Fig. S6b, S7) adopted after (Konjufca et al. 2006).

A gentamicin-free infection of HT-29 infection was done to assess possibility of expression and delivery of Laz (and its derivatives) during an in vitro experimental setup (Fig. 3c). Laz (YCL), NSP4Laz (YCN), and SptPLaz (YCP) were detected in both extracellular fractions, bacterial crude lysates and secreted proteins. Laz (YCL) was the only variant reliably detected in the intracellular-delivered protein fraction. HT-29 was chosen for this test as it carries a mutated p53 gene, an established target for Laz protein.

The uncontrolled bacterial growth associated with the above infection conditions, led to extensive death of host cells (Supplementary Fig. S8), highlighting the need to use an antibiotic (gentamicin) to preserve monolayer integrity.

### In vitro cytotoxicity assays

#### Optimization of steps in infection protocols

Washing steps, media composition, and gentamicin concentrations were optimized in uninfected HT-29 cultures. Higher cell viability was considered desirable for increasing assay sensitivity and minimizing stress-related cell death. Minimal PBS washing, and use of DMEM (instead of PBS) during standard duration of gentamicin treatment (1 h) preserved higher cell viability (Supplementary Fig. S9). Gentamicin up to 200 µg/mL did not adversely affect viability, and 50 µg/mL was adopted for subsequent experiments.

#### Laz-producing strains compared to TetR^−^ control strain

After a standard one-hour treatment of gentamicin to kill extracellular bacteria, a better invasion of MCF-7 cells was observed for YDL (YB1-pET29a-*laz*) compared to YC (YB1-pET29a), at both MOIs 100 (8.29 ± 0.94% vs. 5.62 ± 0.61%; *p* = 0.02) and 1000 (1.5 ± 0.71% vs. 0.7 ± 0.40%) (Supplementary Fig. S10a). The ten-fold increase of bacterial dose significantly reduced invasion %, due to the disproportionate increase of invading bacterial counts (YDL = 1.8-folds; YC = 1.25-folds). This indicates saturation of invasiveness between both MOIs.

Despite the inferiority of invasion capabilities, YC (TetR^−^) significantly outgrew YDL (TetR^+^) in the extracellular compartment at the end of the assay (Supplementary Fig. S10b). This disparity in growth is likely due to the metabolic burden imposed by the constitutive expression of the repressor protein TetR in YDL, and speculatively in the other Laz-derivative producers as well.

Such discrepancy in growth was affected the results of in vitro cytotoxicity assays, especially when gentamicin was removed (after standard one-hour treatment). YC consistently exhibited greater cytotoxicity than YDL regardless of aTc concentration used (Supplementary Fig. S10c). Nevertheless, YDL showed a progressive increase of cytotoxicity with increasing inducer concentration reaching the maximum at 1000 ng/mL aTc. One-way ANOVA revealed a significant difference among the tested conditions (F (9,20) = 7.18, *p* < 0.001), indicating that inducer concentration and strain used have significantly affected cell viability. The difference in cytotoxicity between YC and YDL was significant during pairwise comparisons made independently within each inducer concentration, except for 1000 ng/mL. A similar trend was also observed with different MOIs (Supplementary Fig. S11; 0 µg/mL gentamicin -after the standard one-hour treatment-). The trend could only be reversed with a higher MOI (1000), and a fully maintained gentamicin concentration (50 µg/mL), but the overall effect was remarkably reduced (Supplementary Fig. S11).

#### Laz-producing strains compared to TetR^+^ control strain

**Fig. 4:**
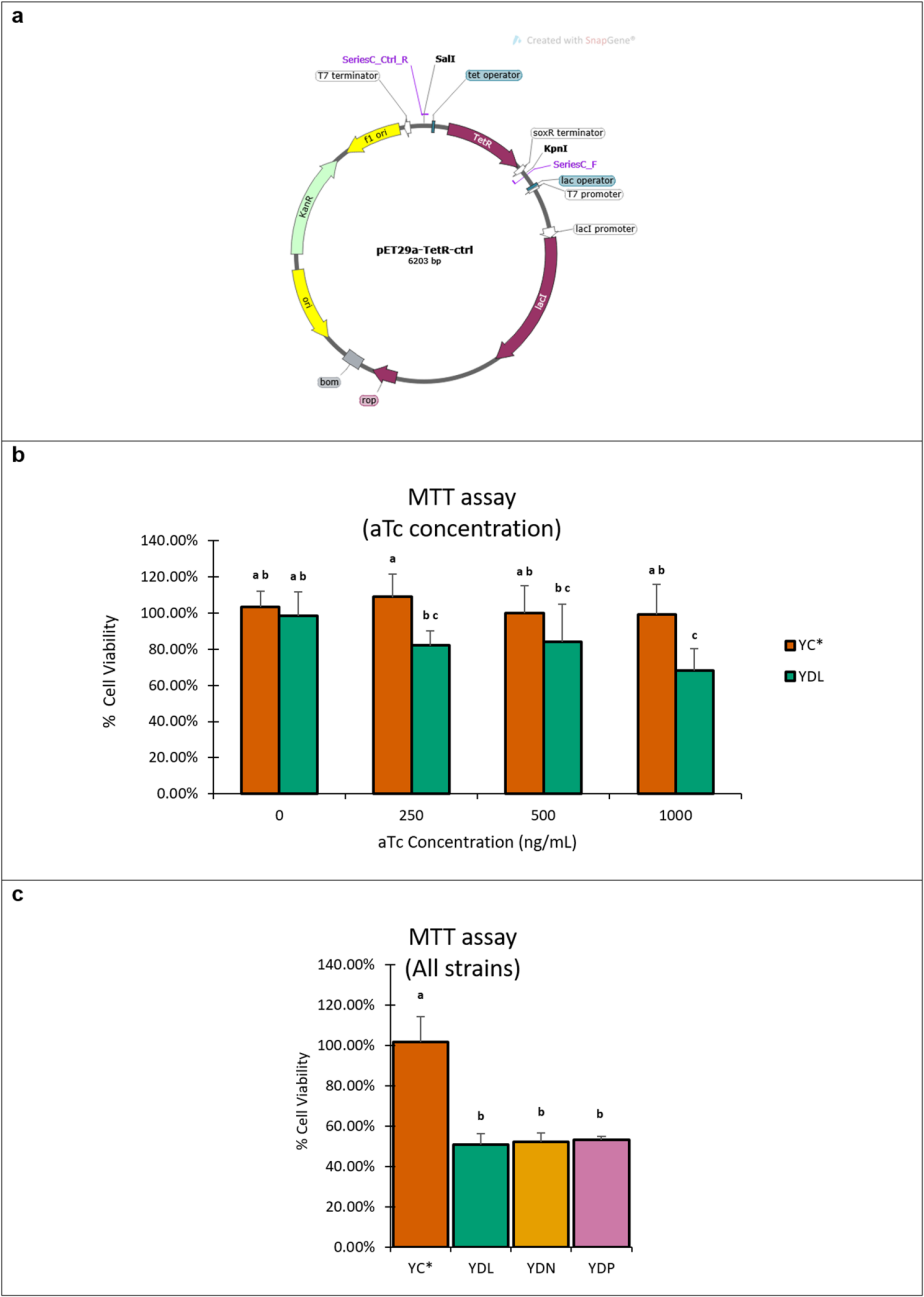
Constructed control (YC*) with matched metabolic burden and associated cytotoxic effect imposed on MCF-7 cells. **a** Vector map for pET29a-*tet*R control plasmid used in YC*. **b** Cell viability of MCF-7 infected with YC* and YDL, under different concentrations of inducer. Standard one-hour treatment of gentamicin (*n* = 9). **c** Cell viability of MCF-7 infected with YC*, YDL, YDN, and YDP. Standard one-hour treatment of gentamicin, aTc concentration = 1000 ng/mL (*n* = 3). Data are presented as mean ± SD. Statistical analysis was performed using one-way ANOVA followed by Tukey’s HSD post-hoc test. Bars sharing at least one common superscript letter are not significantly different (*p* < 0.05)

Due to the unmatched metabolic burden between the control strain and Laz-producing strains, another control strain (YB1-pET29a-*tet*R; YC*) was constructed (Fig. 4a). Cytotoxicity assays run with YC*, under the same experimental conditions, showed a significant change in the outcome (Fig. 4b). A one-way ANOVA revealed a significant difference among the tested conditions (F(7,60) = 7.80, *p* < 0.001). YDL showed higher levels of cytotoxicity than YC* at all induced concentrations, especially with 1000 ng/mL aTc (68.32 ± 12% cell viability), which caused the most prominent reduction in cancer cell viability. Similar to YC, increasing aTc concentration with YC* treatment did not show any significant differences in cell viability indicating absence of inducer-related toxicity on mammalian cell viability. Accordingly, a concentration of 1000 ng/mL aTc was chosen for further in vitro assessments. Another important observation, none of the YC* groups was significant from the uninduced YDL group, indicating that both strains have comparable metabolic loads, growth, and cytotoxic activities in absence of Laz expression.

Further cytotoxicity assays involving all three Laz-producing strains YDL, YDN (YB1-pET29a-*nsp4laz*) and YDP (YB1-pET29a-*sptPlaz*) (Fig. 4c) revealed a similar, and significantly increased response of all three compared to YC* through a one-way ANOVA (F(3,8) = 32.96, *p* < 0.001) followed by post-hoc Tukey’s HSD. The most prominent response was observed for YDL (50.69 ± 5.51%), but was not significantly different from either YDN (52.05 ± 4.69%), or YDP (51.03 ± 3.92%) treatments. 

### In vivo study on antitumor activity

After promising antitumor activity was observed in vitro, an animal study was conducted on Ehrlich Ascitic Carcinoma (EAC) mouse model. Groups included a model untreated group receiving PBS injections, experimental groups comprised of YC* (YB1-pET29a-tetR), YDL (YB1-pET29a-laz), YDP (YB1-pET29a-sptPlaz), and YB1 (parent strain), in addition to two more healthy -non-tumor bearing- (groups for safety assessment purposes, injected with YC* or PBS).

Weights of mice, volumes of tumors were tracked from the day of inoculation until sacrifice, followed by histological examinations of tumor sections (thigh muscle tissue from healthy groups), and morphometric analysis for tumor sections.

#### Tumor growth and volume progression

**Fig. 5:**
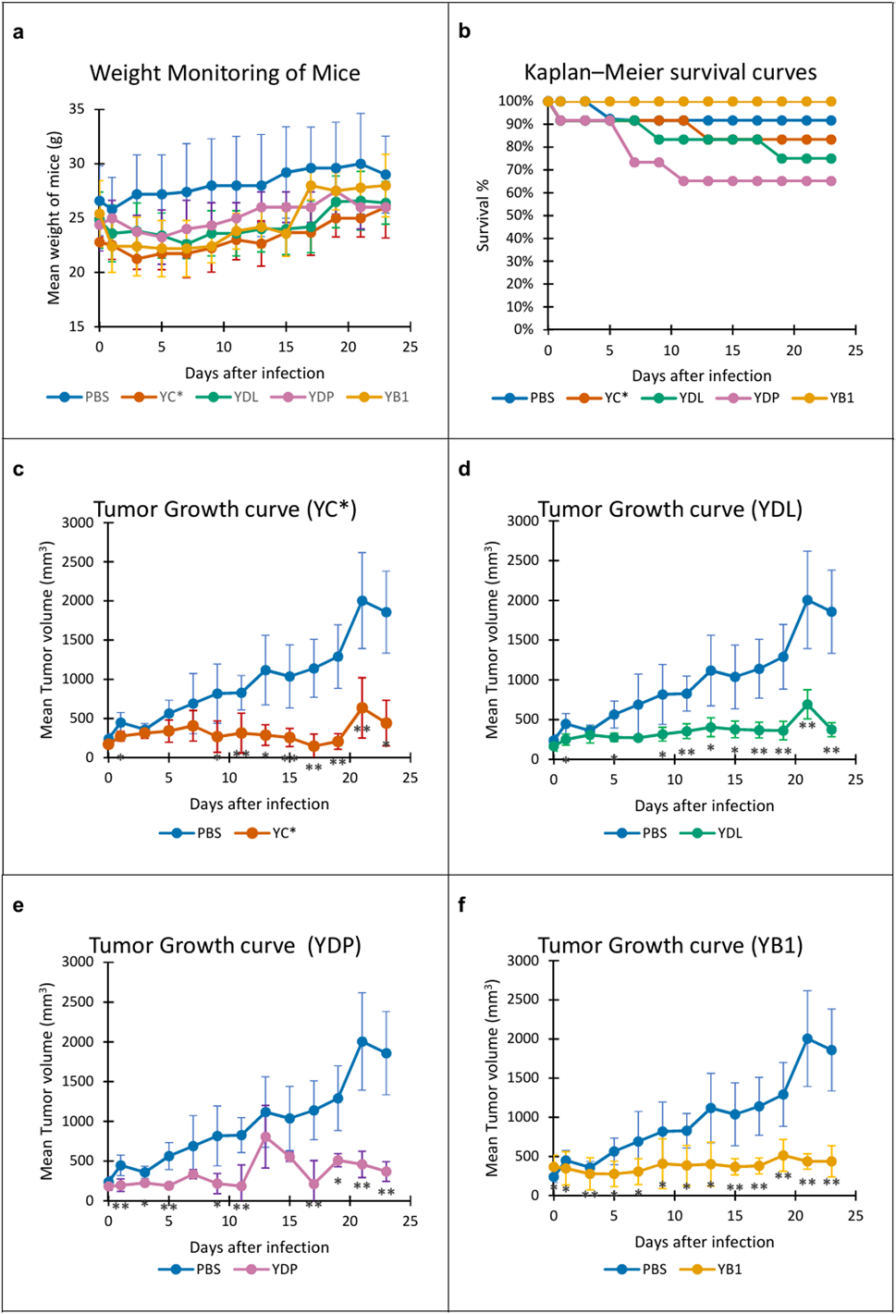
In vivo evaluation of bacterial treatments in the Ehrlich Ascitic Carcinoma (EAC) mouse model. Bacterial treatments showed partial inhibition of tumor growth compared with the model control group.** a** Body weight monitoring of mice over the experimental period. Data are presented as mean ± SD (*n* = 5 mice per group). Statistical analysis was performed using mixed two-way repeated-measures ANOVA. No statistically significant differences were observed after Greenhouse–Geisser correction (*p* = 0.16). **b** Kaplan–Meier survival analysis of tumor-bearing mice following bacterial treatment. Survival curves represent pooled data from two independent experiments (*n* = 13 mice per group). **c–f** Tumor volume growth curves for YC*, YDL, YDP, and YB1 treated groups, respectively, compared with the model control group (PBS). Tumor volumes were measured over time and are presented as mean ± SD (*n* = 5 mice per group). Statistical analysis was performed using mixed two-way repeated-measures ANOVA. Pairwise comparisons between each treatment group and the PBS control at individual time points were performed using Student’s t-test. Asterisks indicate significant differences compared with PBS (*p < 0.05, **p < 0.01)

Tumor development was successfully established in all inoculated animals, with palpable masses detected by day 7 post-inoculation. A Mixed Two-Way Repeated Measures ANOVA was performed to assess the impact of treatments on mouse body weight. No significant treatment-by-time interaction was observed after Greenhouse-Geisser correction (*p* = 0.16), indicating that body weight remained stable across all experimental groups throughout the 23-day study. These results suggest that the treatments were well-tolerated with no evidence of systemic toxicity or significant weight loss (Fig. 5a). Kaplan–Meier survival analysis showed that all bacterial treatments were generally well tolerated, with survival rates ranging from 65% to 100% during the 23-day observation period (Fig. 5b).

Tumor growth (Fig. 5c and f) progressed steadily in the untreated model group (PBS), which showed the largest mean tumor volumes throughout the observation period. Treated groups exhibited clear suppression in tumor growth compared to PBS group. Tumor growth was monitored over time in mice receiving different treatments. A mixed two-way repeated measures ANOVA revealed significant effects of treatment (F(4,20) = 7.91, *p* = 0.00054), time (Greenhouse–Geisser corrected F(3.41,68.22) = 19.54, *p* < 0.000001), and a significant treatment × time interaction (F(13.64,68.22) = 8.91, *p* < 0.000001), indicating that tumor growth trajectories differed between groups. Post-hoc comparisons showed that all treatment groups exhibited significantly reduced tumor volume compared with the PBS control at later time points. Endpoint analysis at day 23 using one-way ANOVA followed by Tukey’s HSD confirmed that all treatments significantly inhibited tumor growth relative to PBS (*p* < 0.05). However, no statistically significant differences were observed between the four treatment groups themselves at this time point (*p* > 0.05).

#### Histological evaluation

**Fig. 6:**
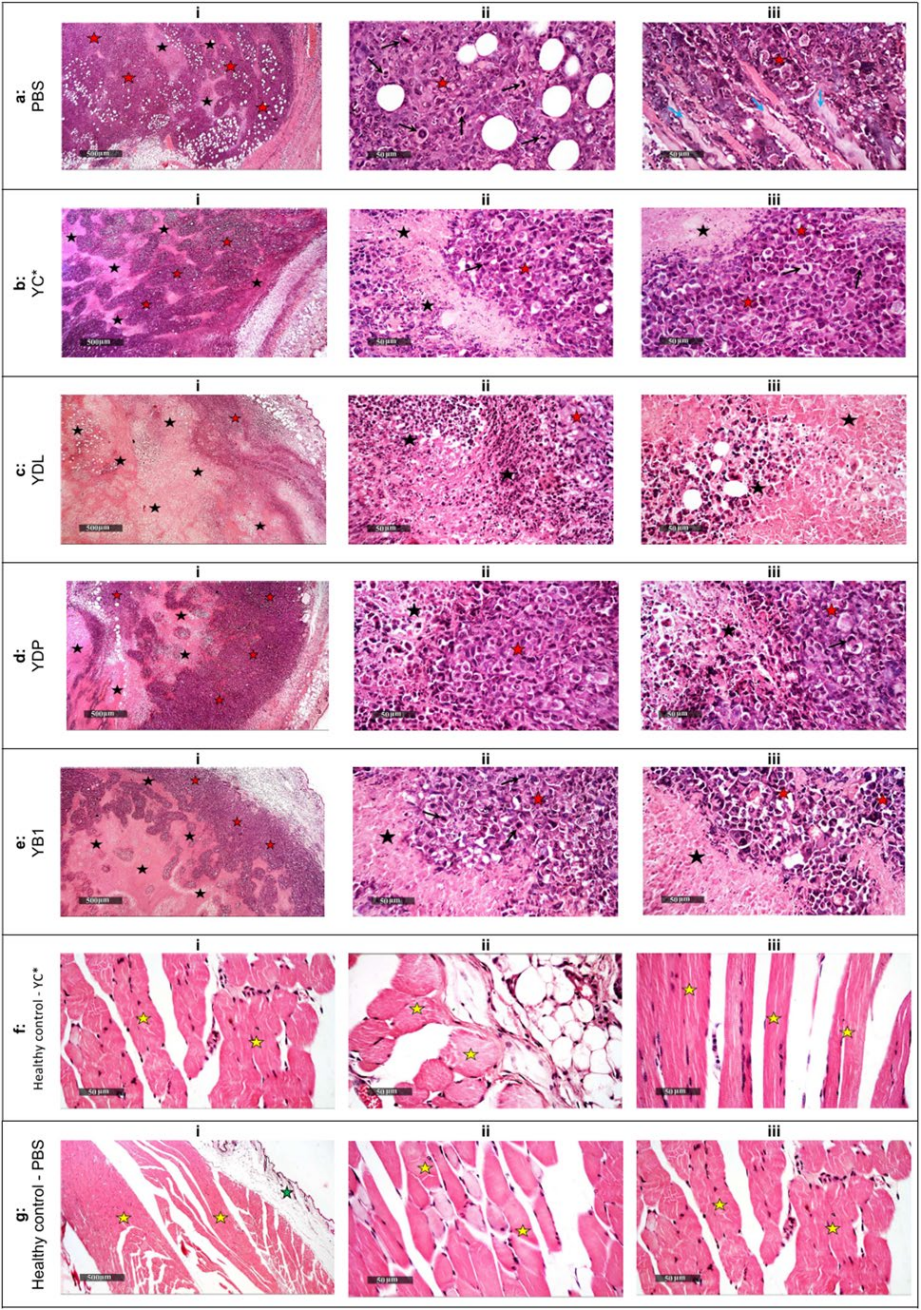
Histopathological features of tumor tissues (**a**–**e**), and of normal thigh muscle tissues (**f**–**g**; safety assessment) . Representative H&E-stained sections showing histological differences among experimental groups. **a** Group 1 (Model, PBS): Tumor sections show dense sheets of pleomorphic basophilic tumor cells with prominent nucleoli (red stars), frequent mitotic figures (black arrows), and minimal focal necrosis (black stars). Tumor cells infiltrate between necrotic muscle fibers (blue arrows). Moderate vascular hyperemia and mild mononuclear inflammatory cell infiltration are also evident. **b** Group 2 (YC*): Tumor sections show a mild to moderate increase in necrotic areas and apoptotic cells (black stars), alternating with persistent sheets of viable tumor cells (red stars). Mitotic figures are infrequent. **c** Group 3 (YDL): Tumor sections exhibit extensive, centrally circumscribed necrosis with marked cellular loss (black stars), with only a thin peripheral rim of viable tumor cells (red stars), indicating strong tumor suppression. **d** Group 4 (YDP): Tumor sections show moderate central necrosis (black stars) with persistence of a moderate outer layer of viable tumor cells (red stars) and occasional mitotic figures (black arrows). **e** Group 5 (YB1): Tumor sections demonstrate greater necrotic areas (black stars) compared with Groups 2 and 4, with variable regions of viable tumor cells (red stars) and occasional mitotic figures (black arrows), surrounded by moderate mononuclear inflammatory cell infiltration. **f** Group 6 (Healthy control - YC*): Tissue sections show normal histological architecture of the outer skin, subcutaneous tissue, and underlying skeletal muscle. Green stars indicate subcutaneous tissue, and yellow stars indicate skeletal muscle fibers. No evidence of tissue damage, necrosis, inflammatory cell infiltration, or abnormal cellular changes was observed. **g** Group 7 (Healthy control - PBS): Sections exhibit comparable normal tissue organization and morphology. Green stars indicate subcutaneous tissue, and yellow stars indicate skeletal muscle fibers. No histopathological alterations were detected. Hematoxylin and eosin (H&E) staining. Scale bar: i = 500 μm (except F-i = 50 μm); ii, & iii = 50 μm.

*Group *1* (Tumor model control - PBS)*. Tumor sections showed dense sheets of viable pleomorphic basophilic tumor cells with prominent nucleoli, frequent mitotic figures, and only minimal focal necrosis. Tumor cells infiltrated between necrotic muscle fibers, and moderate hyperemia of surrounding vasculature was observed with mild mononuclear cell infiltration. These findings are consistent with aggressive tumor growth (Fig. 6a).

*Group *2 (YC*). Tumor sections exhibited a mild to moderate increase in necrotic areas compared with the model group, accompanied by increased numbers of apoptotic cells. However, large areas of viable tumor cell sheets persisted, although mitotic figures were reduced (Fig. 6b).

*Group *3* (YDL).* This group showed the most evident antitumor effect. Sections were characterized by a wide, centrally circumscribed necrotic area with marked cellular loss and only a thin peripheral rim of viable tumor cells. Mitotic figures were rare, indicating strong suppression of tumor cell proliferation (Fig. 6c).

*Group *4* (YDP).* Tumor tissues demonstrated moderate central necrosis with persistence of a moderate outer layer of viable tumor cells. Occasional mitotic figures were observed, suggesting partial but incomplete tumor suppression (Fig. 6d).

*Group *5* (YB*1*)*. Sections showed more extensive necrotic areas than those observed in Groups 2, and 4. However, variable areas of viable tumor cells and occasional mitotic figures were still present. Tumor tissues were surrounded by moderate mononuclear inflammatory cell infiltration (Fig. 6e).

*Group *6 (Healthy control - YC*) and Group 7* (Healthy control - PBS)*. Both groups exhibited normal histological architecture of skin, subcutaneous tissue, and muscle, with no evidence of abnormal cellular changes, necrosis, or inflammatory infiltrates (Fig. 6f and g).

#### Morphometric analysis of necrotic area

**Fig. 7:**
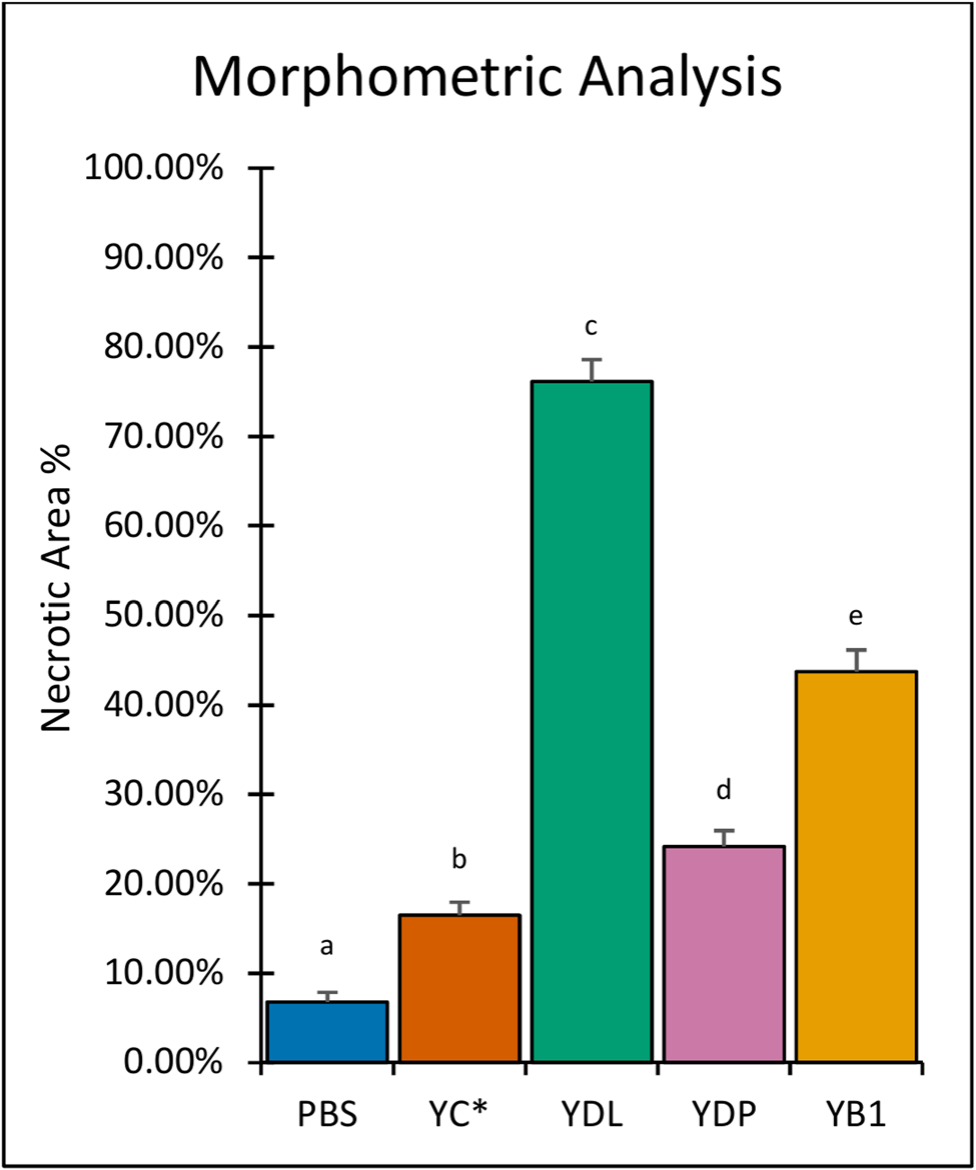
Morphometric analysis of necrotic areas in Ehrlich carcinoma tumor sections following treatment with different bacterial strains. Quantitative assessment of the percentage of necrotic area was performed on H&E-stained tumor sections. Tumors treated with Laz-expressing YB1 strains exhibited a significant increase in necrotic area compared with PBS and YC* controls, with YDL showing the highest extent of tumor necrosis. Data are presented as mean ± SD. Statistical analysis was performed using one-way ANOVA (F (4,25) = 1217.54, *p* < 0.001) followed by Tukey’s HSD post-hoc test. Bars sharing at least one common superscript letter are not significantly different (*p* < 0.05).

Quantitative morphometric analysis (Fig. 7) supported the qualitative histological observations, the indications taken from tumor growth progression, and in vitro assays. A one-way ANOVA revealed a significant difference among the tested conditions (F (4,25) = 1217.54, *p* < 0.001). Group 3 (YDL) exhibited the highest percentage of necrotic tumor area (76 ± 2%) among all treated groups. Group 5 (YB1) showed a higher necrotic area percentage (44 ± 2%) than Groups 2 (YC*, 16 ± 1% ), and 4 (YDP 24 ± 2%), whereas Group 1 (PBS) demonstrated the lowest proportion of necrosis (7 ± 1%). The healthy control Groups 6 and 7 showed no necrotic or pathological changes.

## Discussion

Bacterial tumor-targeted therapy is a promising approach due to their self-replicative nature and selective accumulation in hypoxic tumor microenvironments (Bermudes et al. 2000; Clairmont et al. 2000; Ganai et al. 2011). Among these, *Salmonella enterica* serovar Typhimurium strains such as VNP20009 and YB1 have been extensively studied (Guo et al. 2015; Kasinskas and Forbes 2006; Leschner and Weiss 2010; Li et al. 2017; Ning et al. 2017; Osswald et al. 2015; Wang et al. 2016; Yu et al. 2012, 2015). YB1, in particular, demonstrates strict anaerobic growth and enhanced tumor specificity, supporting its use as a candidate for delivering therapeutic payloads.

In the present study, we focused on evaluating YB1-mediated delivery of of Laz using different secretion signals, and the associated functional cytotoxic effects. Expression of Laz was driven by the chemically inducible Pxyl/tet promoter, and various secretion pathways were tested: the native signal peptide (SPase II) (Gotschlich and Seiff 1987), the NSP4 signal peptide (SPase I) (Han et al. 2017), and Type III secretion signals from SopE and SptP (Konjufca et al. 2006, 2008). Compared with pUC19, the pET29a vector provided higher plasmid stability in YB1 and enabled successful protein expression.

Western blot analyses confirmed expression of Laz, NSP4Laz, and SptPLaz in bacterial culture and during infection of mammalian cells, whereas SopELaz was not expressed. The three constructs exhibited an inducer-dependent increase of Laz band intensity in the crude cell lysates. However, varying secretion patterns were observed. Laz secretion increased with aTc concentration, NSP4Laz displayed time-dependent kinetics, while SptPLaz secretion remained relatively weak. Although the SptPLaz secretion increased under intestinal-mimicking conditions and during interaction with HT-29 cells, the present experiments provide indirect evidence of T3SS-mediated delivery. Direct demonstration of cytoplasmic translocation would require additional assays such as reporter-based translocation systems.

Optimization of infection protocols highlighted critical parameters, including washing strategies, media selection, and gentamicin maintenance. Excessive PBS washing and prolonged PBS exposure significantly reduced cancer cell viability. Furthermore, a standard treatment of gentamicin (50 µg/mL^–1^ h) was found to be necessary to prevent bacterial overgrowth, thereby maintaining integrity of cancer monolayers.

During in vitro infections of MCF-7, it was revealed that YC (YB1-pET29a; *tet*R^−^) had a growth-related advantage over YDL (and other Laz-producing strains), despite having an invasion-related disadvantage. As a result, YC exhibited stronger cytotoxic profile compared to YDL at all inducer concentrations (except at 1000 ng/mL aTc). The consistency of the observation for uninduced YDL, and the lack of leaky expression, as revealed by western blots, pointed out that the metabolic burden was imposed by the constitutive expression of the TetR repressor protein, and not expression of Laz. Restriction of extracellular growth by maintaining gentamicin overcame some differences in growth but the total effect was very limited. These findings underscore the need to balance expression control with vector fitness when designing live bacterial therapeutics.

To test the above hypothesis, a new TetR-expressing control strain (YC* - YB1-pET29a-*tet*R) was constructed, and tested in a replicated experimental setup (as done previously with YC). Compared to YC*, Induced YDL sets showed significantly stronger reductions of MCF-7 viability. Moreover, lack of significant differences between uninduced YDL and every YC* treatments showed that both strains have closely matched growth and baseline activities, which strongly supports the hypothesis. Although the increment of inducer from 250 to 1000 ng/mL was not statistically significant, increasing aTc concentration for YDL infections was accompanied with a consistent increase in response, peaking at 1000 ng/mL. The effect of this inducer concentration was significantly stronger than uninduced YDL and all YC* treatments. Conversely, increasing aTc concentration had no effect on YC* (or YC) treatments, indicating safety of using aTc up to 1000 ng/mL with MCF-7 cells. These results, together with the western blotting data, indicate that the stronger effect observed at 1000 ng/mL aTc is associated with induction-dependent Laz expression, rather than an adverse effect of the inducer itself.

Further assays to test the effect of other constructed strains, YDN (YB1-pET29a-*nsp4laz*), and YDP (YB1-pET29a-*sptPlaz*), and comparing it with YC* and YDL were performed. All three Laz-producing strains exhibited very similar levels of cytotoxicity, and were all significantly stronger than YC*. Although YDL exhibited higher Laz expression levels compared to YDN and YDP, the differences in cytotoxicity observed in MCF-7 cells were modest and not statistically significant. This may reflect differences in protein delivery mechanisms. While YDL primarily relies on surface expression, YDN secretes Laz into the extracellular environment, and YDP is designed to deliver Laz into host cells via the type III secretion system (T3SS). These differences in delivery strategies may influence the effective availability of Laz to target cells despite differences in total protein expression levels. The combined results of the in vitro cytotoxicity experiments supported progression to in vivo studies to further evaluate antitumor activity.

An animal study was conducted as well to assess the antitumor activities of the test strains (YC*, YDL, YDP, and YB1 parent strain). All bacterially treated groups showed reduced tumor growth compared to the model group. Mean weights of mice increased slightly during the study, but without any significant differences between the groups. However, differences in tumor volume progression over time were observed, as tumors of the model group grew significantly more than all other bacterially treated groups. The fluctuations between the four experimental groups were mostly non-significant. Although modest differences in survival were observed between groups, overall survival remained high, suggesting that administration of the engineered bacterial strains was well tolerated under the experimental conditions. The present study did not quantify bacterial colonization levels in tumor and normal tissues. Future studies will include CFU-based biodistribution analyses to determine whether differences in therapeutic outcomes are influenced by bacterial colonization dynamics in addition to Laz-associated effects.

Histopathological examination supported the gross and morphometric findings and demonstrated clear differences in tumor architecture among the experimental groups. Untreated tumor tissues were characterized by densely packed pleomorphic malignant cells, frequent mitotic figures, and only limited spontaneous necrosis, reflecting the aggressive nature of the Ehrlich carcinoma. In contrast, treatment with the bacterial strains induced varying degrees of tumor tissue damage, manifested primarily as expansion of necrotic areas and reduction of viable tumor cell populations. Again aligning with superior protein expression, the most pronounced effect was observed in the YDL-treated groups, where extensive central necrosis with only thin peripheral layers of viable tumor cells was evident. This pattern is associated with reduced tumor viability and structural disruption of tumor tissue. Moderate responses were observed in some intermediate treatment groups, which showed mixed areas of necrosis and residual viable tumor tissue. Importantly, muscle tissues obtained from non-tumor control groups preserved normal histological architecture without inflammatory infiltration or cellular atypia, supporting the local safety of the bacterial treatment. Collectively, these findings support that the observed effects are associated with true structural destruction of tumor tissue rather than non-specific tissue damage. Although YDL treatment produced a larger necrotic area within tumors, the reduction in tumor volume was not significantly different from that observed with the YC* or YB1 strains. This may reflect the strong intrinsic antitumor activity of YB1 under the current experimental conditions, which could partially mask additional therapeutic effects mediated by Laz expression. Optimization of treatment parameters, including bacterial dose and administration schedule, may further clarify the contribution of Laz to antitumor efficacy in future studies.

The present study highlights the complexity and multifactorial considerations involved in the design, development, and evaluation of bacterial tumor-targeting systems. Several key parameters were optimized to establish a functional system with controlled protein expression and delivery, including the use of a metabolically matched control strain (YC*), which enabled more accurate interpretation of cytotoxicity results. The observed induction-dependent effects and differences between constructs emphasize the importance of balancing expression control, delivery strategy, and bacterial fitness when designing engineered bacterial platforms.

The distribution and expression of Laz protein within tumor tissues were not examined. Future studies employing immunohistochemistry or protein detection assays will be necessary to characterize intratumoral localization and persistence following administration. Additionally, the development of a stable, chemical inducer-free, and antibiotic-free expression system, through the use of alternative promoters and chromosomal integration strategies, would further improve the applicability of this platform.

## Conclusion

This study demonstrates the successful inducible expression and export of Laz and its derived variants in *S. Typhimurium* YB1. Among the constructs, the native Laz system showed higher expression and export levels compared with the derived variants. Laz-expressing strains exhibited induction-dependent reductions in cell viability and were associated with observable changes in tumor morphology in vivo.

Overall, this work provides a functional evaluation of Laz delivery using the YB1 system and highlights key design considerations for engineered bacterial delivery platforms. 

## Supplementary Information

Below is the link to the electronic supplementary material.

Supplementary Material 1.

## Data Availability

No datasets were generated or analysed during the current study.
