# Supplementary material for: In vitro and in vivo antitumor activity of Laz-expressing Salmonella Typhimurium YB1 via optimized secretion and regulation
Source: AMB Express. 2026 May 15;16:54. doi: 10.1186/s13568-026-02057-x (PMC13179408; doi:10.1186/s13568-026-02057-x)
Supplement: Supplementary file 1 — Supplementary Material 1. [file 13568_2026_2057_MOESM1_ESM.pdf]

Table S 1: DNA sequences of the codon-optimized *laz* gene for *Salmonella*. The native *laz* coding sequence is shown with the original signal peptide highlighted in bold. In the constructs listed below, the native signal peptide region was replaced with alternative secretion signal sequences (*nsp4*, *sopE*, and *sptP*), also highlighted in bold, which were fused in-frame to the laz coding region to generate the corresponding secretion variants used in this study. All sequences are presented in the 5′→3′ orientation.

**Name**	**Sequence 5'--> 3'**	**Description**
laz	**ATGAAAGCTTATCTTGCCCTTATTTCGGCAGCAGTCATTGGGTTGGCAGCTTGT**TCACAG<br>GAACCAGCTGCCCCTGCCGCGGAAGCTACGCCAGCAGCCGAAGCGCCAGCGAGTGAAGCA<br>CCCGCGGCGGAAGCCGCACCAGCAGATGCTGCGGAGGCTCCAGCGGCAGGGAACTGCGCC<br>GCGACAGTTGAAAGCAACGACAATATGCAATTCAATACTAAAGATATTCAAGTGAGCAAG<br>GCTTGCAAGGAGTTTACGATTACTTTAAAACATACAGGGACTCAACCAAAGGCATCGATG<br>GGACATAATTTGGTAATTGCAAAGGCTGAAGACATGGATGGTGTATTCAAAGACGGAGTA<br>GGAGCCGCGGACACAGACTATGTGAAACCCGACGATGCACGGGTTGTGGCTCATACAAAA<br>TTAATCGGAGGTGGGGAGGAGGCGTCTCTTACGTTGGATCCCGCCAAACTGGCTGACGGC<br>GAGTACAAATTCGCCTGTACTTTTCCAGGGCATGGTGCACTTATGAACGGTAAGGTCACC<br>CTGGTCGATTAA	Neisserial *laz* gene, codon optimized for *Salmonella;* **SPaseII-mediated secretion.**
nsp4-	**ATGAAGAAGATTACGGCAGCCGCTGGTTTACTTCTGTTGGCCGCTCAACCAGCCATGGCT**	**SPaseI-mediated secretion.**
sopE-	**ATGACAAAAATAACTTTATCTCCCCAGAATTTTAGAATCCAAAAACAGGAAACCACACTA<br>CTAAAAGAAAAATCAACCGAGAAAAATTCTTTAGCAAAAAGTATTCTCGCAGTAAAAAAT<br>CACTTCATCGAATTAAGGTCAAAATTATCGGAACGTTTTATTTCGCATAAGAACACTGAG<br>TCTTCTGCAACACACTTTCACCGAGGAAGCGCATCTGAGGGCCGGGCAGTGTTGACAAAT**	**T3SS-mediated secretion.**
sptP-	**ATGCTAAAGTATGAGGAGAGAAAATTGAATAATTTAACGTTGTCTTCGTTTTCAAAAGTT<br>GGTGTGTCGAATGATGCCCGACTTTATATTGCTAAGGAAAATACTGATAAGGCATATGTT<br>GCGCCTGAAAAATTTTCGTCAAAAGTATTAACCTGGCTTGGAAAAATGCCGTTATTTAAA<br>AACACTGAAGTGGTGCAAAAACATACGGAAAATATCAGAGTACAGGACCAAAAGATTTTA<br>CAGACATTTCTCCATGCACTAACGGAAAAATATGGGGAAACAGCGGTTAATGACGCACTG<br>TTAATGTCCCGTATAAATATGAACAAACCCCTTACCCAACGTTTAGCAGTGCAGATCACG<br>GAGTGTGTAAAAGCTGCTGACGAAGGGTTTATAAACCTTATTAAGAGCAAGGATAATGTT**	**T3SS-mediated secretion.**

Table S 2: List of the primer sequences used in this work and their use. All sequences are presented in the 5′→3′ orientation.

	**Name**	**Sequence 5'--> 3'**	**Use**
1	Primer 1	GATCCTCTAGAGTCGACCTGCAGTACAGCTCCAATGGAGATC	Forward primer for amplification of promotor fragment from pNZ-sg1594 and cloning to pUC19.
2	Primer 2	TTCCAATTCCTCCTCATC	Reverse primer for amplification of promotor fragment from pNZ-sg1594 and cloning to pUC19.
3	Primer 3	GATGAGGAGGAATTGGAAAATG	Forward primer for amplification of synthesized *laz* fragments and cloning to pUC19.
4	Primer 4	TACGCCAAGCTTGCATGC	Reverse primer for amplification of synthesized *laz* fragments and cloning to pUC19.
5	Primer 5 (SeriesC_F1)	GACAGCCCAGATCTGGGTACCTACAGCTCCAATGGAGATC	Forward primer for cloning of promotor + *laz* fragments into pET29a.
6	Primer 6 (SeriesC_R1)	GCGGCCGCAAGCTTGTCGACATCGACCAGGGTGACC	Reverse primer for cloning of promotor + *laz* fragments into pET29a with C-terminal His-tag.
7	Primer 7 (SeriesD_R)	GCGGCCGCAAGCTTGTCGACTTAATCGACCAGGGTG	Reverse primer for cloning of promotor + *laz* fragments into pET29a without C-terminal His-tag.
8	Primer 8 (SeriesC_Ctrl_R)	GTGCGGCCGCAAGCTTGTCGACTTATTCCAATTCCTCCTCATC	Reverse primer for cloning of *tet*R into pET29a to generate plasmid for YC* control strain.

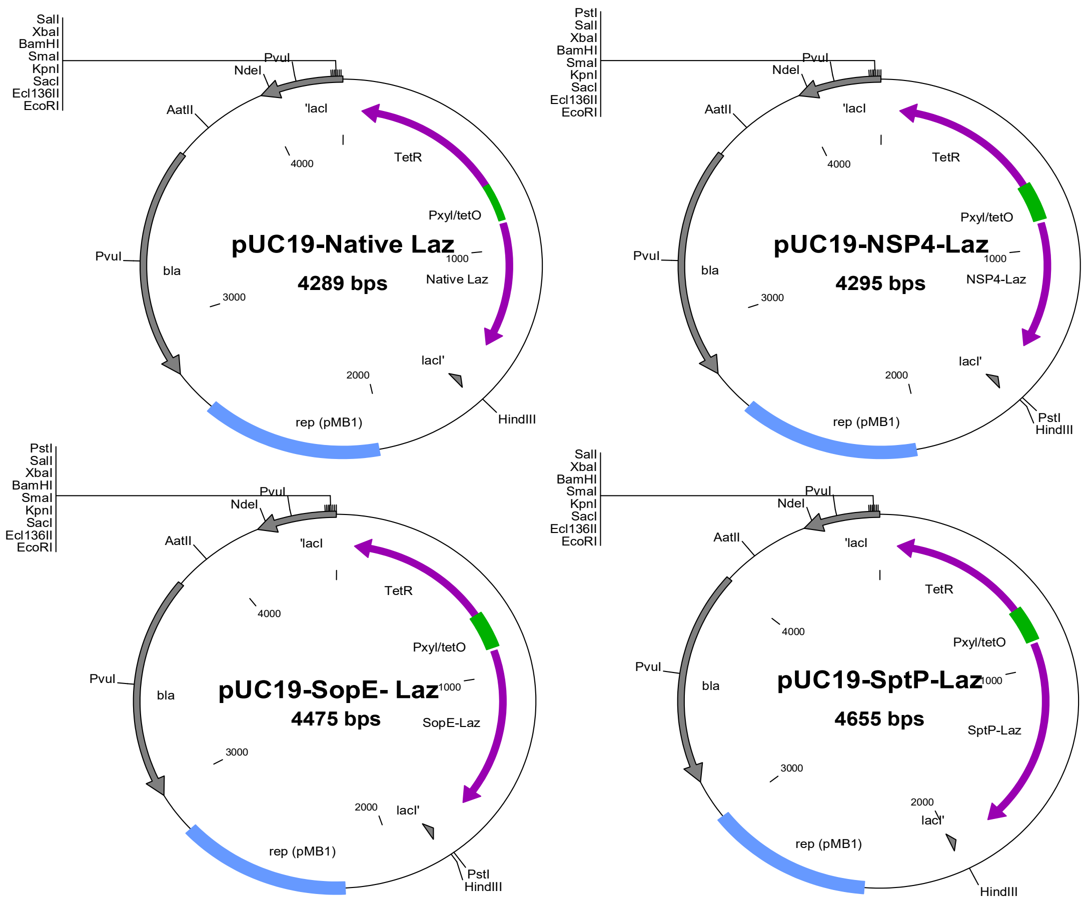

Figure S 1: *laz* variants cloned in pUC19. Images created by Clone manager 5.

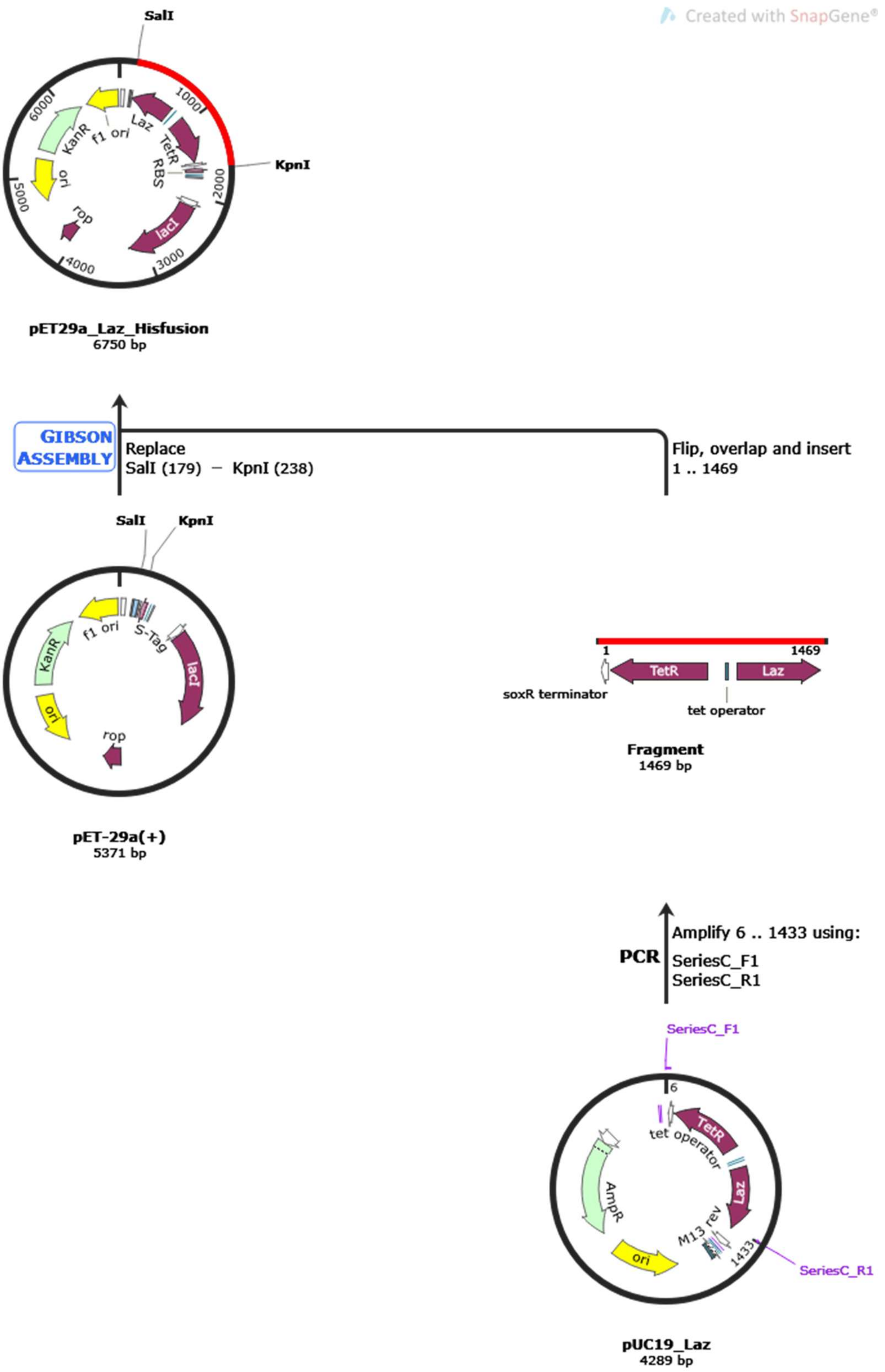

Figure S 2: Cloning workflow of pET29a-laz-His based plasmid constructs. Images generated by SnapGene.

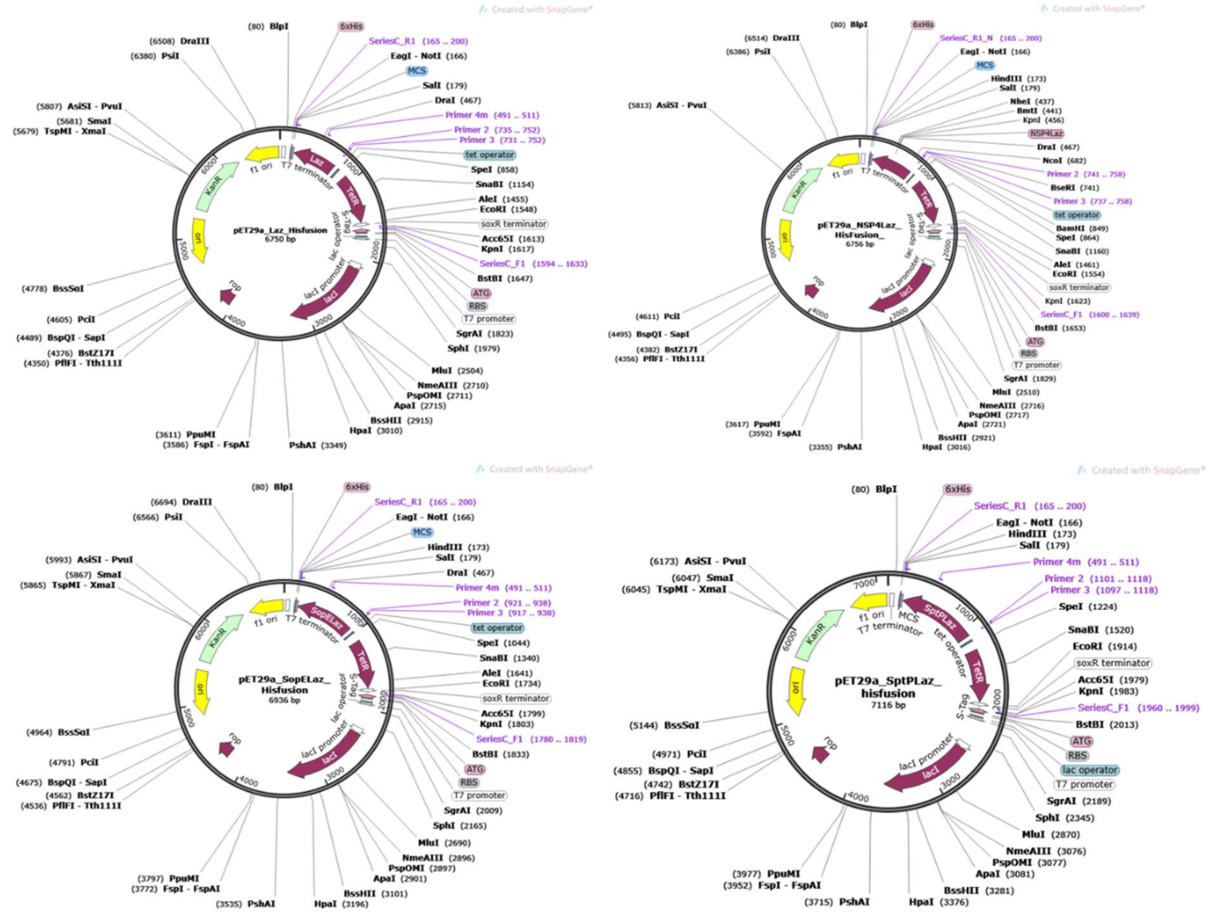

Figure S 3: laz variants cloned in pET29a. Images created by SnapGene.

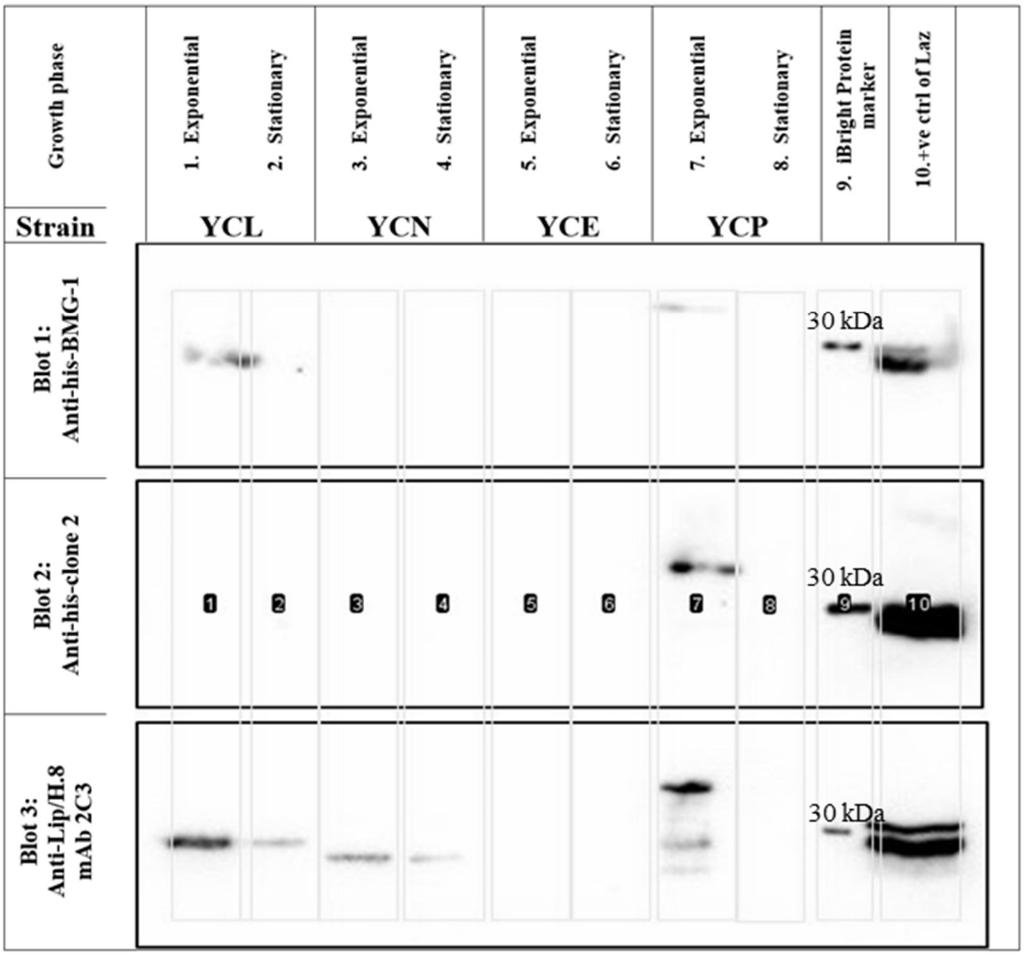

Figure S 4: Western blots of induced YB1 cultures harboring pET29-*laz-his* (YCL) in lanes 1&2, pET29a-*nsp4laz-his* (YCN)-his in lanes 3&4, pET29a-*sopElaz-his* (YCE) in lanes 5&6, and pET29a-*sptPlaz-his* (YCP) in lanes 7&8; in the exponential and stationary phases, respectively. Blots 1, 2 and 3 were treated with anti-His-BMG-1, anti-His-clone 2 or anti-Lip/H.8 mAb 2C3 as the primary antibody, respectively.

Laz, NSP4Laz, and SptPLaz, were detected both in exponential and stationary phases by anti-Lip/H.8 mAb 2C3, but only Laz, and SptPLaz in exponential phase could be detected by anti-His antibodies, which highlighted that anti-Lip/H.8 mAb 2C3 was more sensitive to Laz and its variants than two different types of anti-his antibodies, and was therefore used for subsequent western blots.

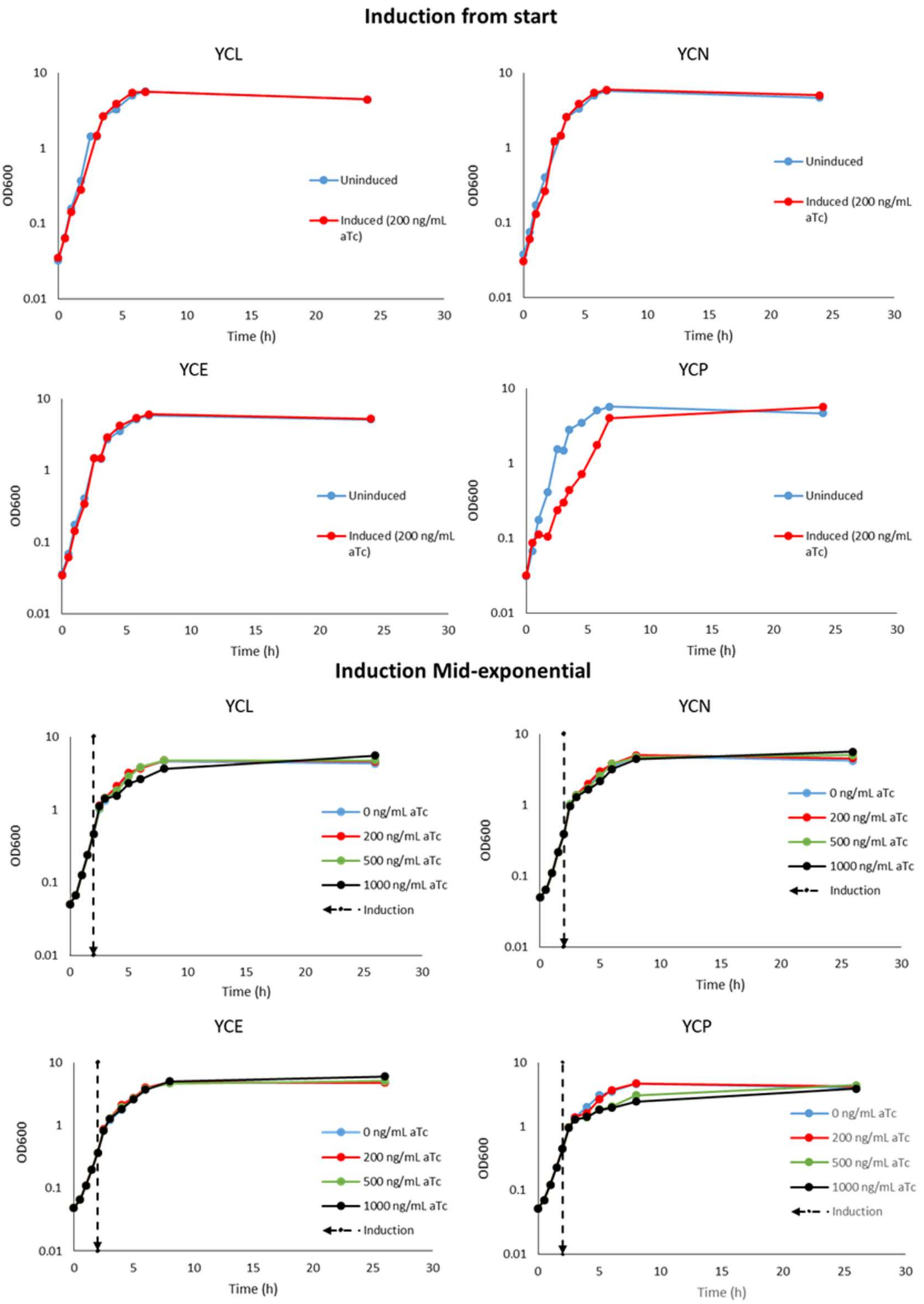

Figure S 5: Growth of YB1-pET29a-*laz-his* (YCL), YB1-pET29a-*nsp4laz-his* (YCN), YB1-pET29a-*sopElaz-his* (YCE), and YB1-pET29a-*sptPlaz-his* (YCP) with different induction strategies/ concentrations

Induction mid-exponential phase showed a clearer concentration dependent hindrance of growth, which was later correlated with protein expression as well.

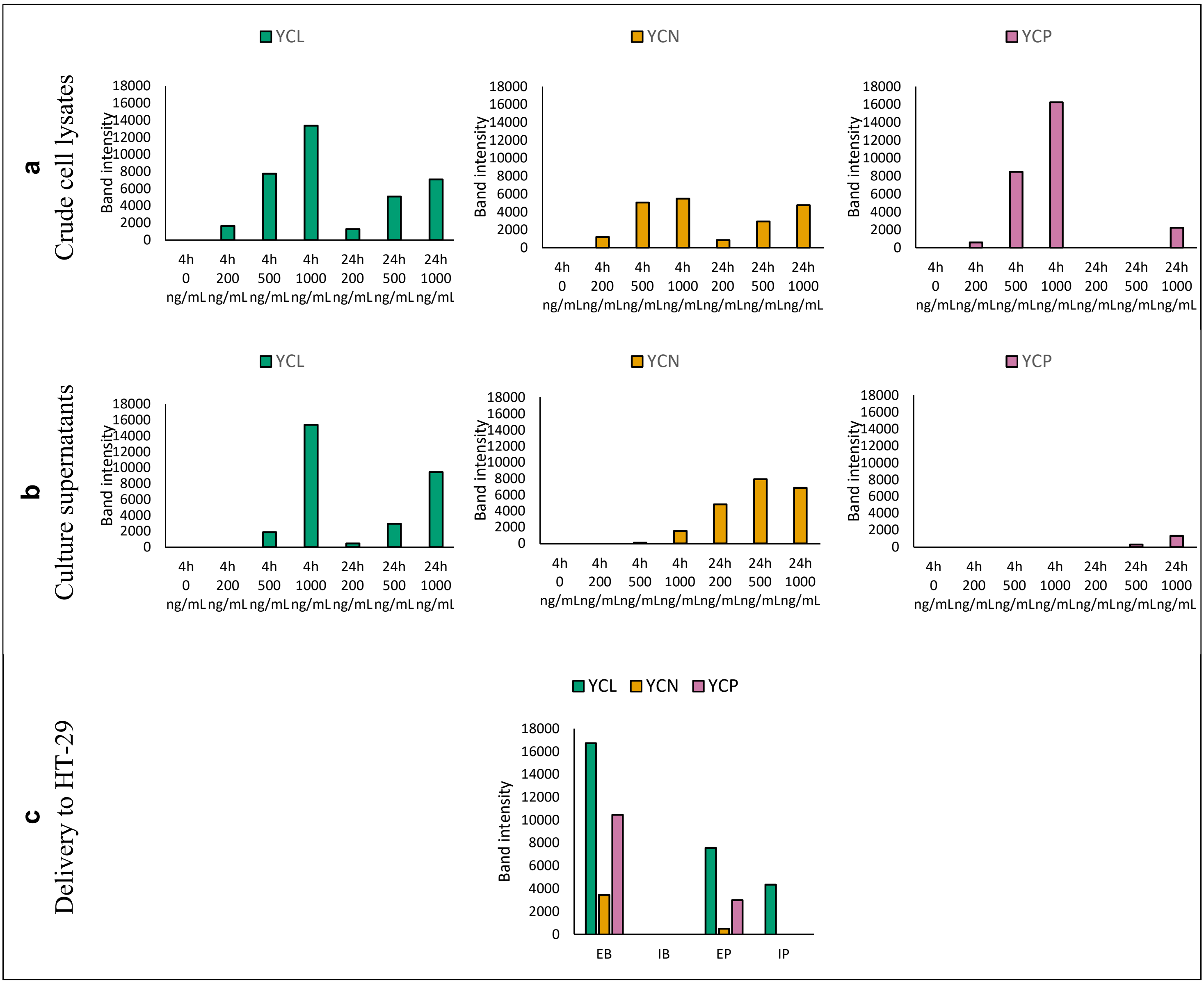

Figure S 6: **Densitometric analysis of Laz western blot bands.**
Band intensities were quantified using ImageJ software and are presented as raw peak intensity values obtained from the corresponding western blot lanes.

(a) Densitometric analysis of Laz detected in crude bacterial cell lysates under different induction conditions. Prior to sample preparation, bacterial cultures were normalized to identical OD_{600} values to ensure comparable bacterial biomass loading across lanes.

(b) Densitometric analysis of Laz detected in culture supernatants, representing secreted protein levels. Supernatant volumes corresponding to equivalent whole-culture OD_{600} values were analyzed to allow comparison of secretion between strains.

(c) Densitometric analysis of Laz following delivery to HT-29 cells. Four fractions were analyzed: extracellular bacterial cell lysate (EB), intracellular bacterial cell lysate (IB), extracellular secreted protein (EP), and intracellularly-delivered protein fraction (IP).

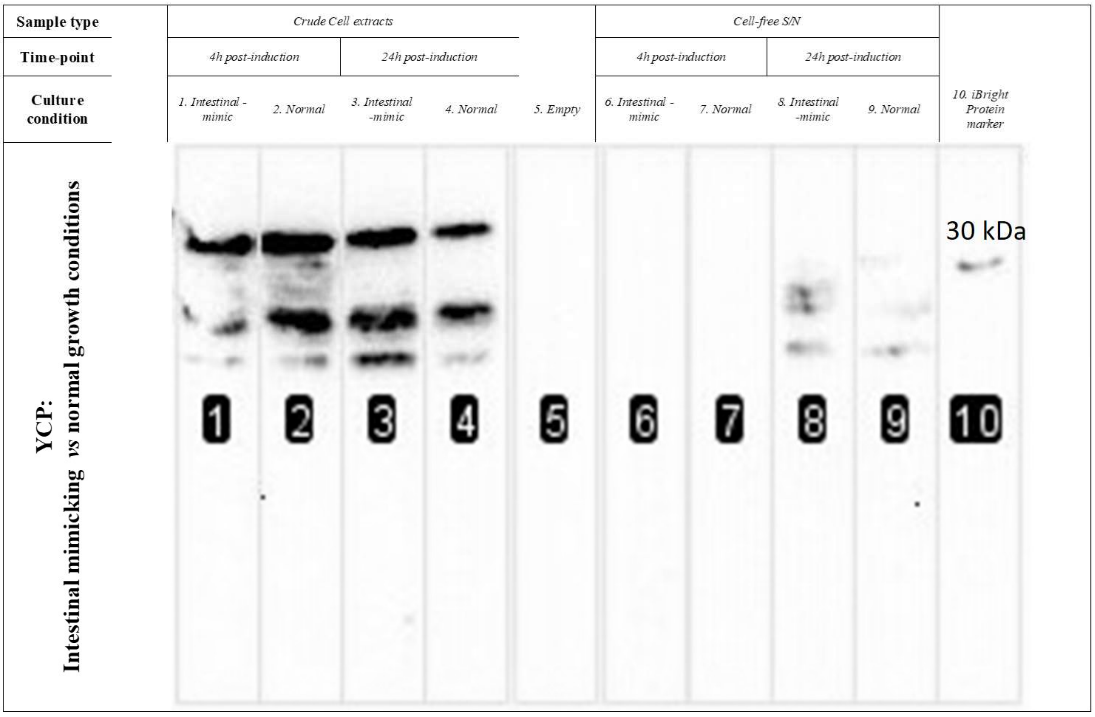

Figure S 7: Western blot to demonstrate SptPLaz expression and secretion under intestinal-mimicking (LB 300mM NaCl, little aeration) or normal growth conditions. Crude cell-lysate samples collected after 4h (lanes 1,2) and 24h (lanes 3,4) of induction. Cell-free S/N samples collected after 4h (lanes 6,7) and 24h (lanes 8,9) of induction.

Conditions mimicking intestinal environment has improved export of SptPLaz.

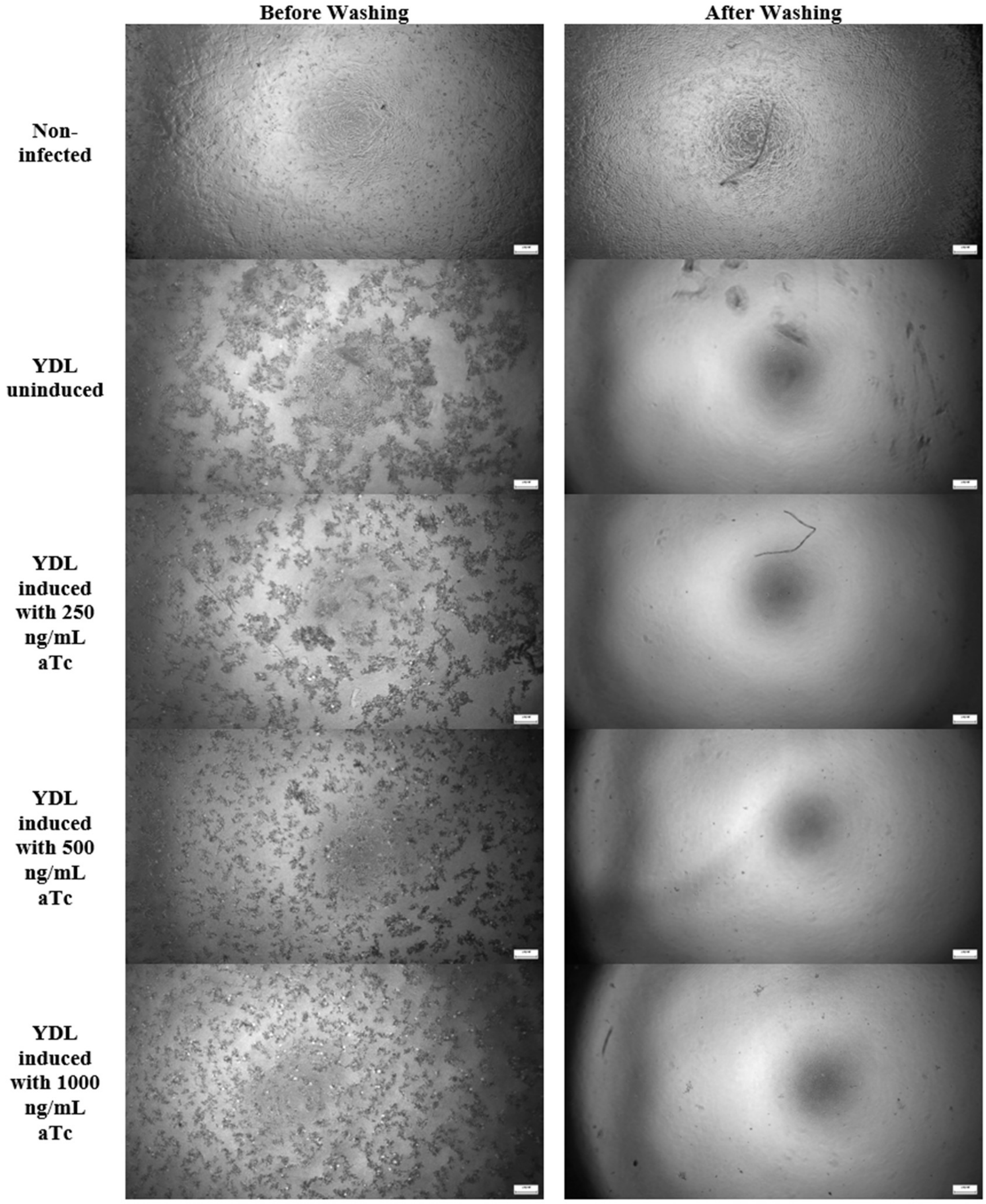

.

Figure S 8: Microscopic images (40x) of MCF-7 after 24h of infection by YB1-pET29a-*laz* in absence of gentamicin, using different inducer concentrations.

Use of gentamicin is important to avoid bacterial overgrowth and excessive mammalian cell death that will mask the effect of the therapeutic proteins under investigation

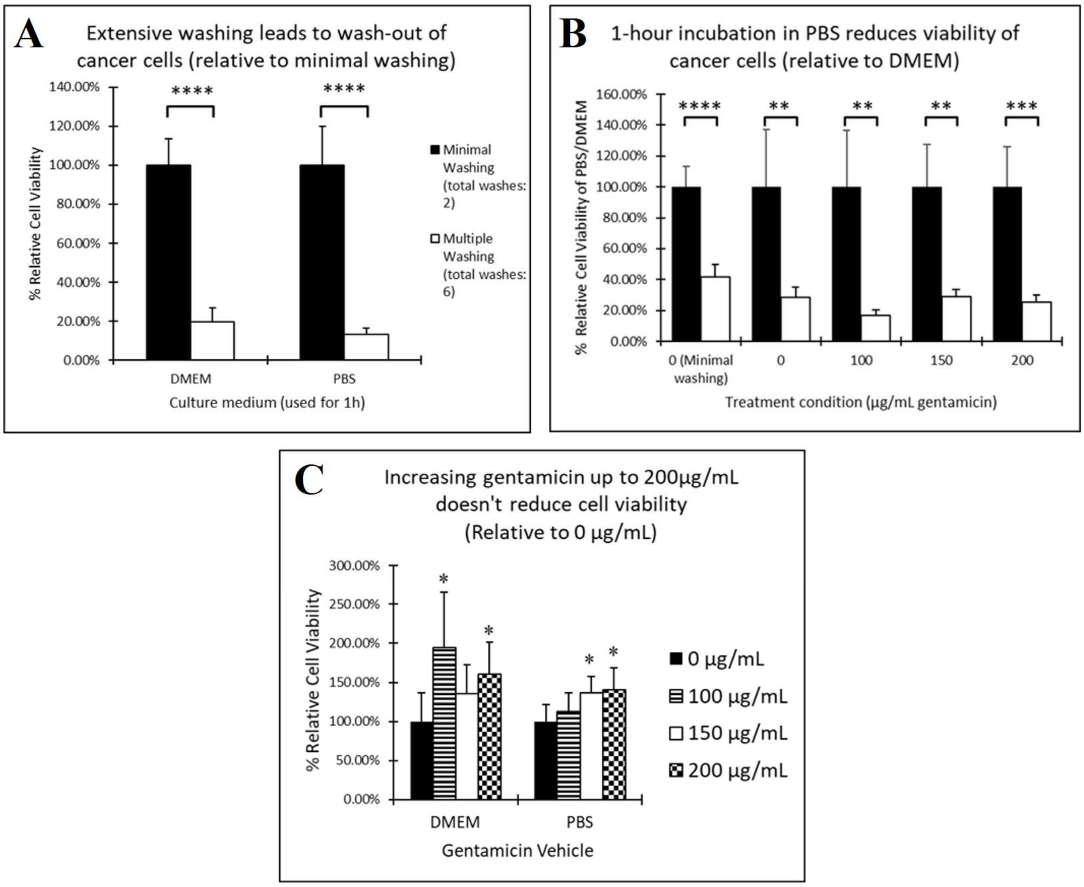

Figure S 9: Assessment and optimization of the experimental conditions for cytotoxicity in the absence of bacterial infection. (A) Effects of extensive washing with PBS on HT-29 cell viability. (B) Effect of one-hour incubation in PBS on HT-29 cell viability. (C) Effect of the gentamicin concentration on HT-29 cell viability. *Data are presented as mean ± SD (n = 3). Pairwise comparisons to corresponding control were evaluated independently using Student's t-test. Asterisks indicate significant differences compared with YC (*p < 0.05, **p < 0.01, ***p < 0.001, ****p < 0.001).*

Extensive washing steps, and use of PBS for incubation with gentamicin significantly affect the viability of mammalian cells for reason irrelevant to infection by bacteria. Meanwhile, gentamicin use is safe up to 200 µg/mL.

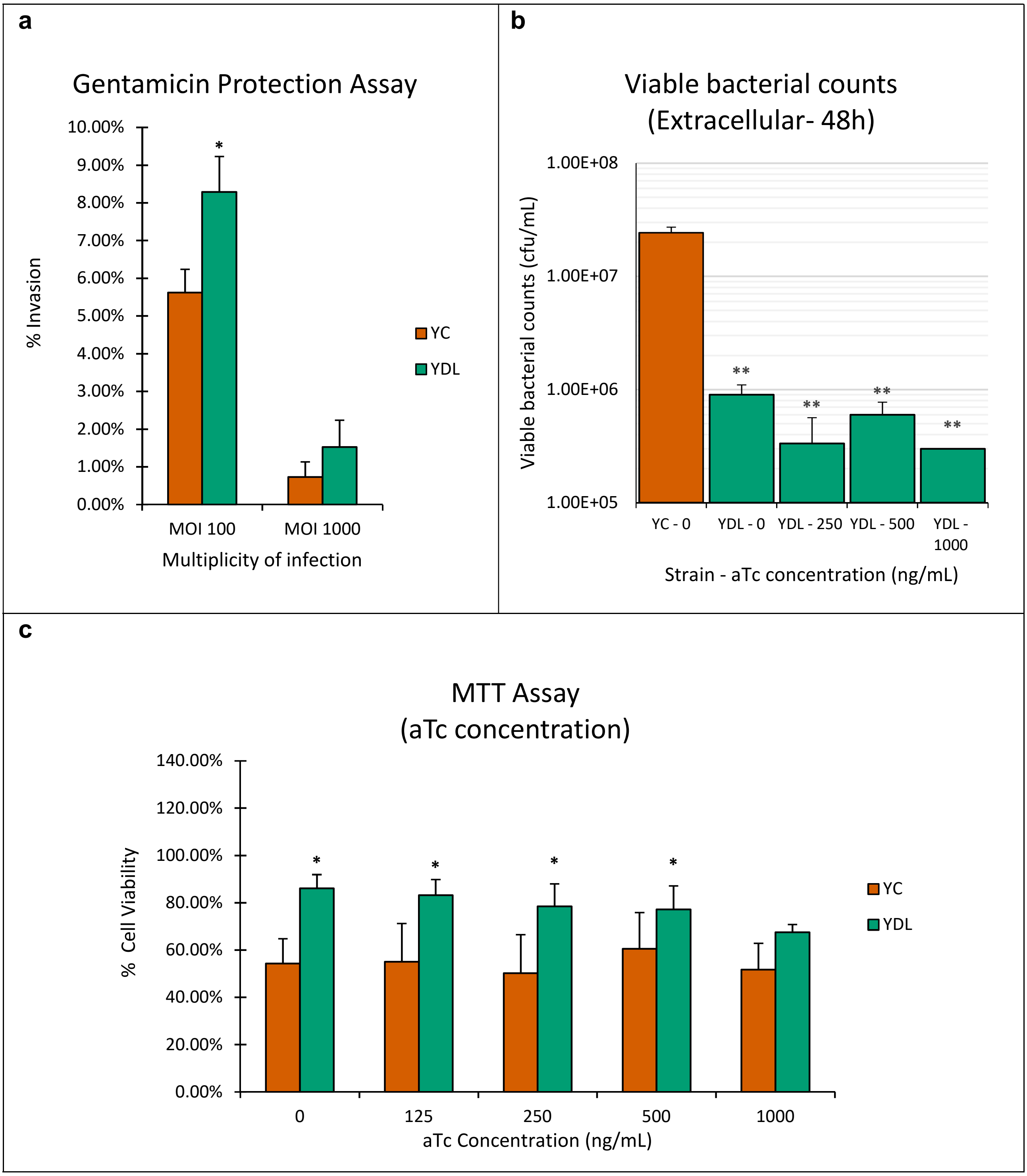

Figure S 10: **Infection of MCF-7 with YC and YDL strains.**
Differences in invasion and growth of YB1-pET29a (YC) and YB1-pET29a-*laz* (YDL) during infections of MCF-7 affect cytotoxicity results.
(a) Invasion of MCF-7 by YC and YDL strains. *Data are presented as mean ± SD (n = 3). Pairwise comparisons to corresponding control (YC) were evaluated independently using Student's t-test. Asterisks indicate significant differences compared with YC (*p < 0.05, **p < 0.01).*
(b) Viable extracellular bacterial counts at 48 h post-infection and standard one-hour treatment of gentamicin. *Data are presented as mean ± SD (n = 3). Pairwise comparisons to corresponding control (YC) were evaluated independently using Student's t-test. Asterisks indicate significant differences compared with YC (*p < 0.05, **p < 0.01).*

(c) Effect of different concentrations of inducer (aTc) on cytotoxicity imposed by YC and YDL on MCF-7 with standard one-hour treatment of gentamicin. *Data are presented as mean ± SD (n = 3). Overall differences among conditions were evaluated using one-way ANOVA (F(9,20) = 7.18, p < 0.001). Bars sharing at least one common superscript letter are not significantly different (p<0.05) as evaluated by Tukey's HSD post-hoc analysis.*

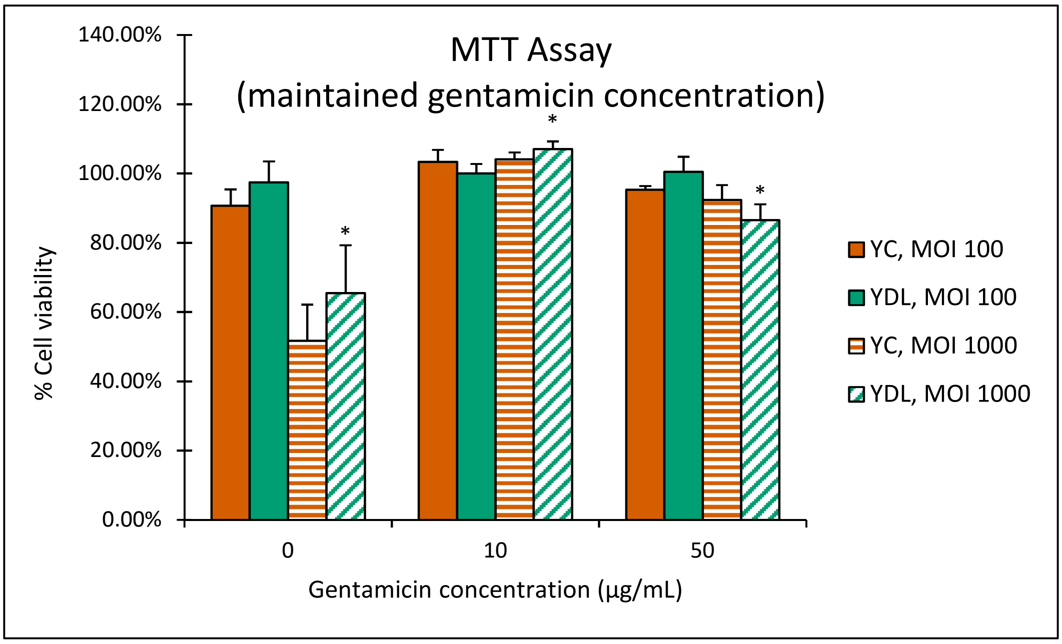

Figure S 11: Effect of removing, reducing (10 µg/mL), or maintaining full concentration of gentamicin (50 µg/mL), after standard one hour treatment, on cell viability of MCF-7 infected with YC and YDL. *Data are presented as mean ± SD (n = 3). Pairwise comparisons to corresponding control (YC) were evaluated independently using Student's t-test. Asterisks indicate significant differences compared with YC (*p < 0.05, **p < 0.01).*

YC causes more cytotoxicity than YDL under all conditions, except with a higher MOI and full maintenance of gentamicin.
